# Alternative splicing is an FXRα loss-of-function mechanism and impacts energy metabolism in hepatocarcinoma cells

**DOI:** 10.1016/j.jbc.2024.108022

**Published:** 2024-11-26

**Authors:** Manon Garcia, Hélène Holota, Angélique De Haze, Jean-Paul Saru, Phelipe Sanchez, Edwige Battistelli, Laura Thirouard, Mélusine Monrose, Gérard Benoit, David H. Volle, Claude Beaudoin

**Affiliations:** 1 Université Clermont Auvergne, CNRS UMR6293, INSERM U1103, iGReD Team-Volle, Clermont-Ferrand, France; 2 Université de Rennes 1, CNRS UMR6290, INSERM U1305, IGDR, Rennes Cedex, France; 3 Centre de Recherche en Nutrition Humaine d’Auvergne, Clermont-Ferrand, France

**Keywords:** nuclear receptor, alternative splicing, gene silencing, metabolism, hepatocellular carcinoma cells

## Abstract

Farnesoid X receptor α (FXRα, *NR1H4*) is a bile acid-activated nuclear receptor that regulates the expression of glycolytic and lipogenic target genes by interacting with the 9-*cis*-retinoic acid receptor α (RXRα, *NR2B1*). Along with cofactors, the FXRα proteins reported thus far in humans and rodents have been observed to regulate both isoform (α1-4)- and tissue-specific gene expression profiles to integrate energy balance and metabolism. Here, we studied the biological functions of an FXRα naturally occurring spliced exon 5 isoform (FXRαse5) lacking the second zinc-binding module of the DNA-binding domain. We demonstrate spliced exon 5 FXRα expression in all FXRα-expressing human and mouse tissues and cells, and that it is unable to bind to its response element or activate FXRα dependent transcription. In parallel, this spliced variant displays differential interaction capacities with its obligate heterodimer partner retinoid X receptor α that may account for silencing of this permissive dimer for signal transduction. Finally, deletion of exon 5 by gene edition in HepG2 cells leads to FXRα loss-of-function, increased expression of LRH1 metabolic sensor and CD36 fatty acid transporter in conjunction with changes in glucose and triglycerides homeostasis. Together, these findings highlight a novel mechanism by which alternative splicing may regulate FXRα gene function to fine-tune adaptive and/or metabolic responses. This finding deepens our understanding on the role of splicing events in hindering FXRα activity to regulate specific transcriptional programs and their contribution in modifying energy metabolism in normal tissues and metabolic diseases.

Farnesoid X receptor α (FXRα, *NR1H4*) is a member of the nuclear receptor (NR) superfamily of ligand-dependent transcription factors that operates as a heterodimer with the retinoid X receptor α (RXRα). Similar to most NRs, FXRα retains a DNA-binding domain (DBD) coupled through a flexible linker, to a ligand-binding domain (LBD), that serves as a transcriptional switch for ligand-dependent recruitment of coregulatory proteins and transcriptional modulation of FXRα target genes (reviewed in (1)). FXRα is highly expressed in enterohepatic tissues such as the liver and intestine, and plays a key role in maintaining cholesterol and bile acid levels as well as in regulating fatty acid and glucose metabolism (2, 3, 4). Besides these metabolic functions, many researchers have highlighted the relevance of FXRα signaling in other biological processes such as steroidogenesis, germ cell fate determination, and male reproduction (5, 6, 7).

Owing to the variety of molecular responses induced by FXRα signaling under physiological and pathological conditions, several efforts have been made to understand the role of NR in regulating a broad transcriptional program to promote ligand-dependent functions, following FXRα/RXRα heterodimerization to FXRα response element (FXRE). As for the other NRs, two zinc finger motifs allow FXRα to interact with its response element which contains two copies of a consensus AGGTCA-like DNA sequence directly reiterated (DR), inverted (IR), or everted (ER), with various inter half-site spacing. Although the FXRα/RXRα heterodimer binds mostly to the consensus IR-1 (IR spaced with one base pair) sequence, the complex also binds to and activates several other FXREs in conjunction with transcriptional coactivators or corepressors that coordinate gene activation or repression following posttranslational modifications (PTMs) of histones and non-histone proteins (8, 9).

In humans and rodents, four biologically active variants have been described so far from a single *NR1H4* locus (10, 11). These distinct isoforms differ in both tissue- and species-specific expression and ligand-dependent transcriptional activities and arise from an alternatively spliced 12-bp extension (amino acid sequence: MYTG) of the DBD in conjunction with two alternative promoters located in the first and third exons of the gene (Fig. 1*A* and (12)). These transcript variants denominated FXRα1 through α4 differ in their activating function domain (AF-1) at the N terminus and the hinge region, immediately adjacent to the DBD. This MYTG insert was observed to affect FXRα isoform ability to bind specific DNA sequences in the promoter region of FXRα target genes with an impact on transcriptional activation (11). Moreover, most metabolic effects regulated by FXRα in mouse and human liver cells were recently reported to be regulated by the FXRα2 isoform, exclusively bound to specific DNA sequences corresponding to everted and IR motifs (13, 14). This finding indicates that cell-specific patterns of FXRα isoforms may be involved in the differential FXRα target gene responses to their activation.

**Figure 1:**
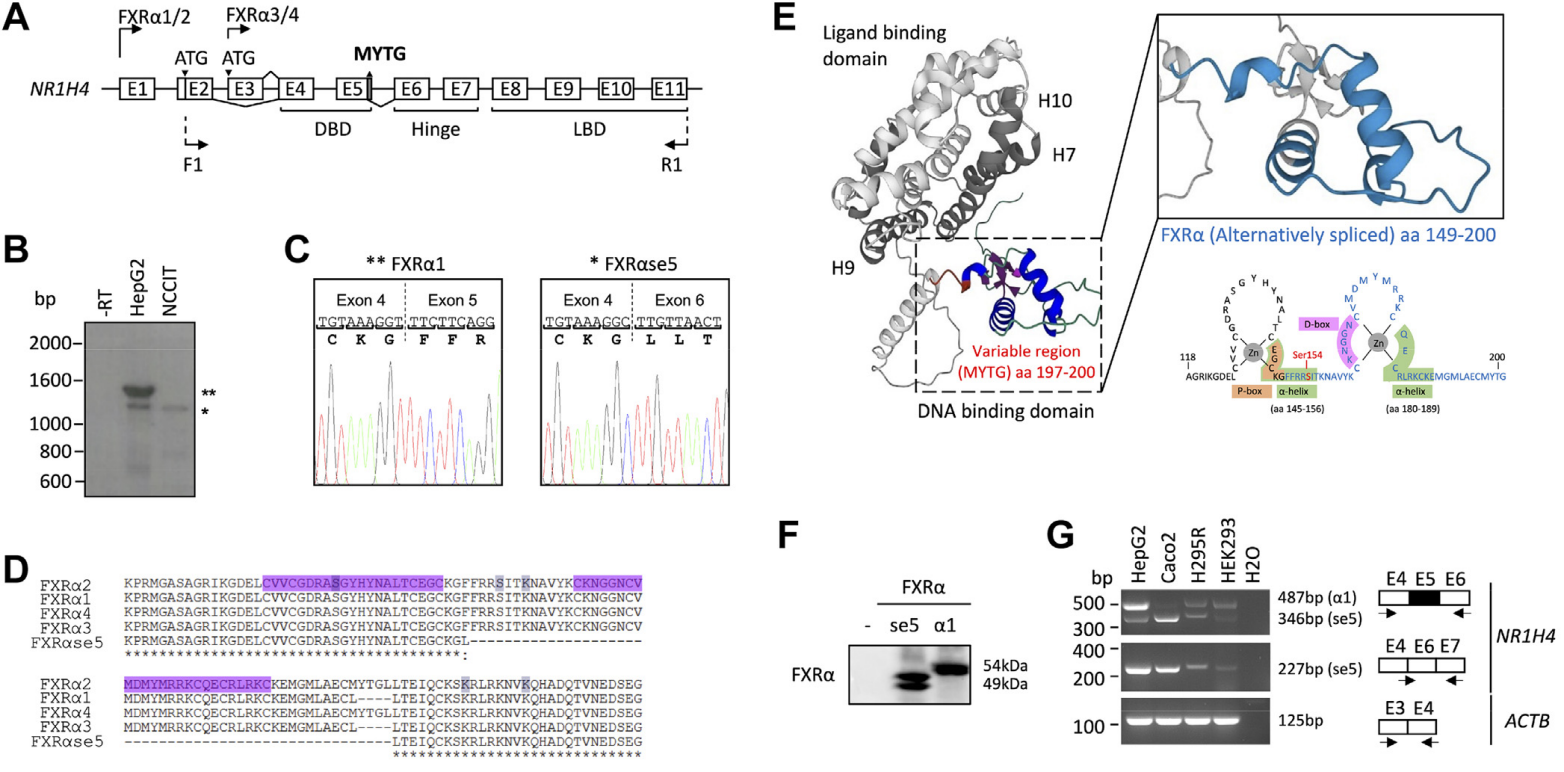
**Identification of a novel alternative splice variant in the human *NR1H4* locus.***A*, schematic representation of human *NR1H4* gene locus with 11 exons and 10 introns. FXRα1/2 and FXRα3/4 are transcribed from exon 1 and exon 3, respectively. The 12-bp insertion is located at the 3′ end of exon 5. Alternative splicing between exon 5 and 6 produces the forms that contain (FXRα2, 4) or do not contain (FXRα1, 3) the 12-bp insert (amino acid sequence: MYTG). Position of the forward (F1) and reverse (R1) primers are indicated. *B*, RT-PCR amplification of FXRα1 and FXRαse5 isoforms from HepG2 and NCCIT cell lines. *C*, sequencing of the RT-PCR amplified fragments from *B*. Chromatograms of FXRα1 and FXRαse5 (*left* and *right* panels, respectively) reveal that the splice variant lacks the entire exon 5. *D*, alignment of FXRα1-α4 and FXRαse5 sequences between exons 4 and 6 (amino acid sequence 111–226). Zinc finger 1 and 2 are highlighted. *E*, AlphaFold prediction modeling of human FXRα1/2 (with MYTG) and magnification of the variable region containing the amino acid sequence of two zinc fingers within the DBD (alternative exon 5-skipping is depicted in blue). Localization of Ser^154^ (*red*) and extension of the proximal-box (P-box, *orange*), distal-box (D-box, *pink*), and two α-helices (*green*) are indicated. FXRα dimer interface composed of helices H10, H9, and H7 are indicated in *gray*. *F*, *In vitro* translation of plasmids encoding FXRα1 and FXRαse5, followed by Western blotting with anti-FXRα antibody. *G*, total RNA isolated from cell line models (1. HepG2, liver; 2. Caco2, colon; 3. NCI-H295R, adrenal; and 4. HEK293, kidney) were analyzed by RT-PCR using primers (labeled with *arrows*) on specific exons and/or at exon-exon junctions. RT-PCR products were separated and bands are marked based on the predicted sizes of the alternatively spliced variants shown beside each gel. Water (lane 5) was used as negative control. DBD, DNA-binding do main; FXRα, farnesoid X receptor α; FXRαse5, spliced exon 5 FXRα.

In the present study, we characterized a naturally occurring spliced variant of FXRα, which harbors an exclusion of exon 5 encoding the second zinc finger of the DBD. The frequency of exon 5 skipping in human (normal and tumor) cell lines and tissues as well as in different mouse tissues suggests that it is a legitimate event with potential biological relevance in modulating FXRα transcriptional activity. Here, we propose alternative splicing of the DBD as a novel inhibitory mechanism leading to FXRα loss-of-function. Notably, FXRα inhibition by alternative splicing may adapt energy metabolism in hepatocarcinoma cells by increasing mRNA expression of the metabolic NR Liver Receptor Homologue 1 (LRH-1) and CD36 fatty acid transporter. This mechanism may regulate metabolic pathways and shed light on events triggering liver diseases and their progression.

## Results

### Identification and cloning of a novel FXR**α** spliced variant in human cells and tissues

**Figure 2:**
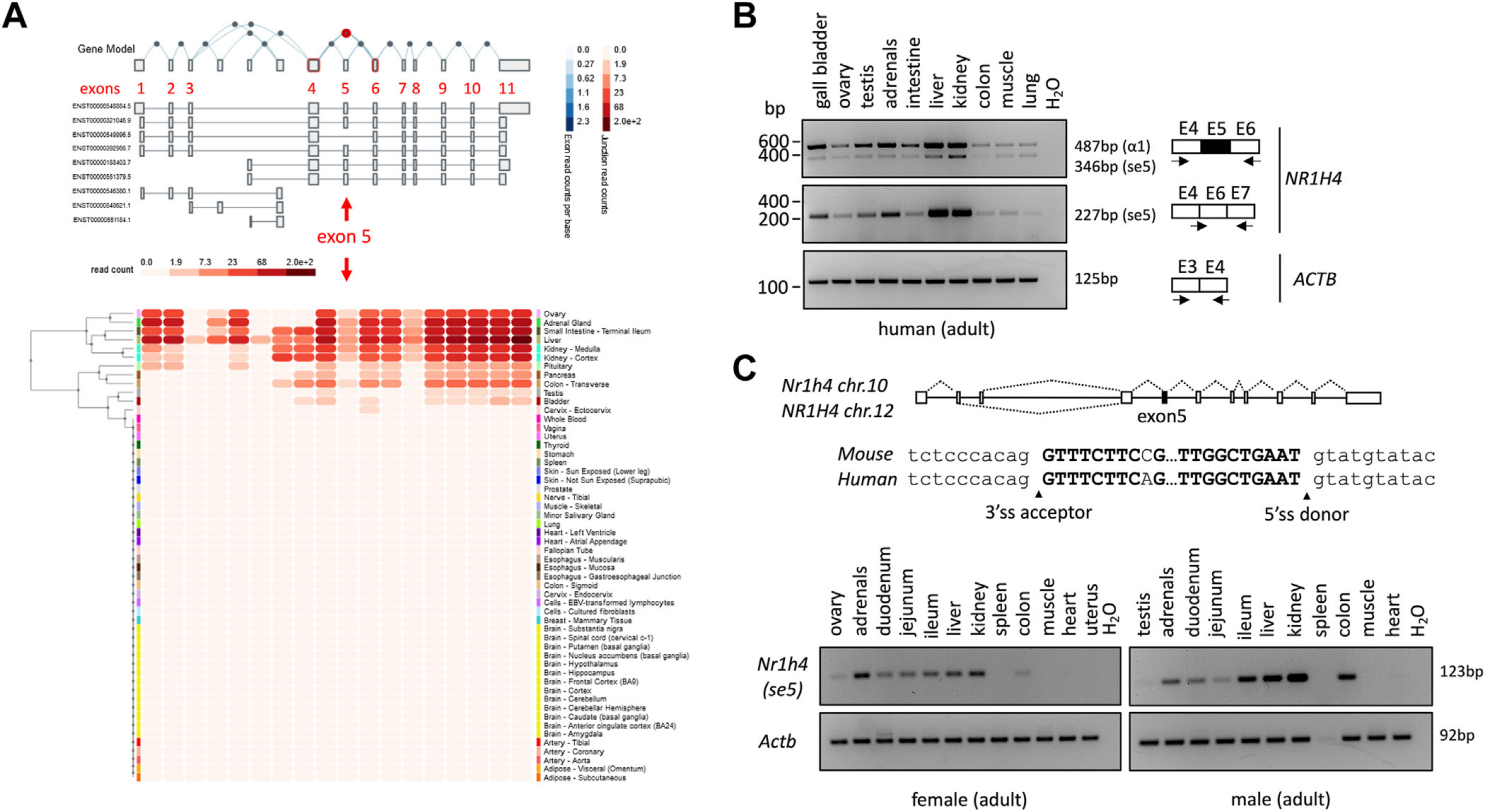
**Analysis of FXRα alternative splicing and frequency of exon 5-skipping in human and mouse tissues.***A*, FXRα exon 5 splicing in different human tissues according to transcriptomic sequencing (RNA-seq) data from the Genome-Tissue Expression portal (GTEx). *B* and *C*, representative gel images of three independent RT-PCR assays for the FXRα isoforms in human (*B*) and murine (*C*) tissues. Alignment of mouse *Nr1h4* gene sequence with human consensus 3′ splice acceptor (3′ss) and 5′ splice donor (5′ss) sites sequences across the intron 4, exon 5, and intron 5 splice sites of *NR1H4* (C, *upper* panel). RT-PCR products were separated and bands are marked based on the predicted sizes of the alternatively spliced variants depicted beside each gel. *Actinb* was used as a reference gene. All sequences for forward and reverse primers (labeled with *arrows*) are available in Table 1. FXRα, farnesoid X receptor α.

While cloning FXRα from cell lines using PCR, we observed a shorter fragment amplifying from HepG2 and NCCIT cells (Fig. 1*B*). Further sequencing of this PCR fragment identified an FXRα splice variant lacking the entire exon 5 of the *NR1H4* gene transcript (Fig. 1*C*). We called this new variant spliced exon 5 FXRα (FXRαse5), which aligned with the FXRα1 to α4 isoforms (referred to as FXRα1−/+ and FXRα2−/+) known thus far; however, it lacked the second zinc finger of the DBD although the fusion of exon 4 and 6 preserved the normal FXRα ORF resulting in a truncated protein (from aa 149–200), which was revealed using *in vitro* translation of the FXRαse5 transcript followed by Western blotting (Fig. 1, *D*–*F*). As FXRαse5 transcript was detectable in model cell lines of tumorigenesis (Fig. 1*G*) including the liver (HepG2), colon (Caco2), adrenal (NCI-H295R), and kidney (HEK293), we speculated whether this novel isoform may be observed in normal human tissues. As depicted in Fig. 2*A*, publicly available RNA sequencing (RNA-seq) data in the Genotype-Tissue Expression portal (GTEx; https://gtexportal.org/home/) supported the evidence of exon 5-skipping in enterohepatic and extra enterohepatic tissues in humans. This finding is further strengthened by the RT-PCR expression profiling of the FXRαse5 isoform that revealed a strong accumulation in the human liver, kidney, adrenals, and gallbladder, and to a lesser extent in the intestine, colon, testis, and ovary (Fig. 2*B*). Alignment of the human *NR1H4* exon 5 intronic boundary regions with the mouse orthologous *Nr1h4* revealed a high degree of conservation for the 3′- (acceptor) and 5′- (donor) splice sites (Fig. 2*C* upper panel); we hypothesized that exon 5-skipping may generate a homologous splice variant in mice. RT-PCR assays in different male and female mice tissues confirmed that alternative splicing of the second zinc-finger of mouse Fxrα DBD (11) is a common event as we were able to detect an equivalent spliced isoform in the liver, kidney, ileum, colon, adrenals, and gonads in both male and female adult mice (Fig. 2*C*, lower panel). Moreover, a circadian clock oscillation is maintained for FXRαse5 mRNA in C57BL/6 male mice liver (Fig. S1). Thus, this splicing event that occurred in human cell types and tissues and the conservation in mice suggests that this naturally occurring splice variant, lacking a portion of the DBD, may not transduce the same ligand-dependent and/or ligand-independent signaling cascade as the WT receptor for eliciting specific changes in gene expression with different metabolic outcomes.

### Exon 5-skipping disrupts FXR**α** gene regulatory function

**Figure 3:**
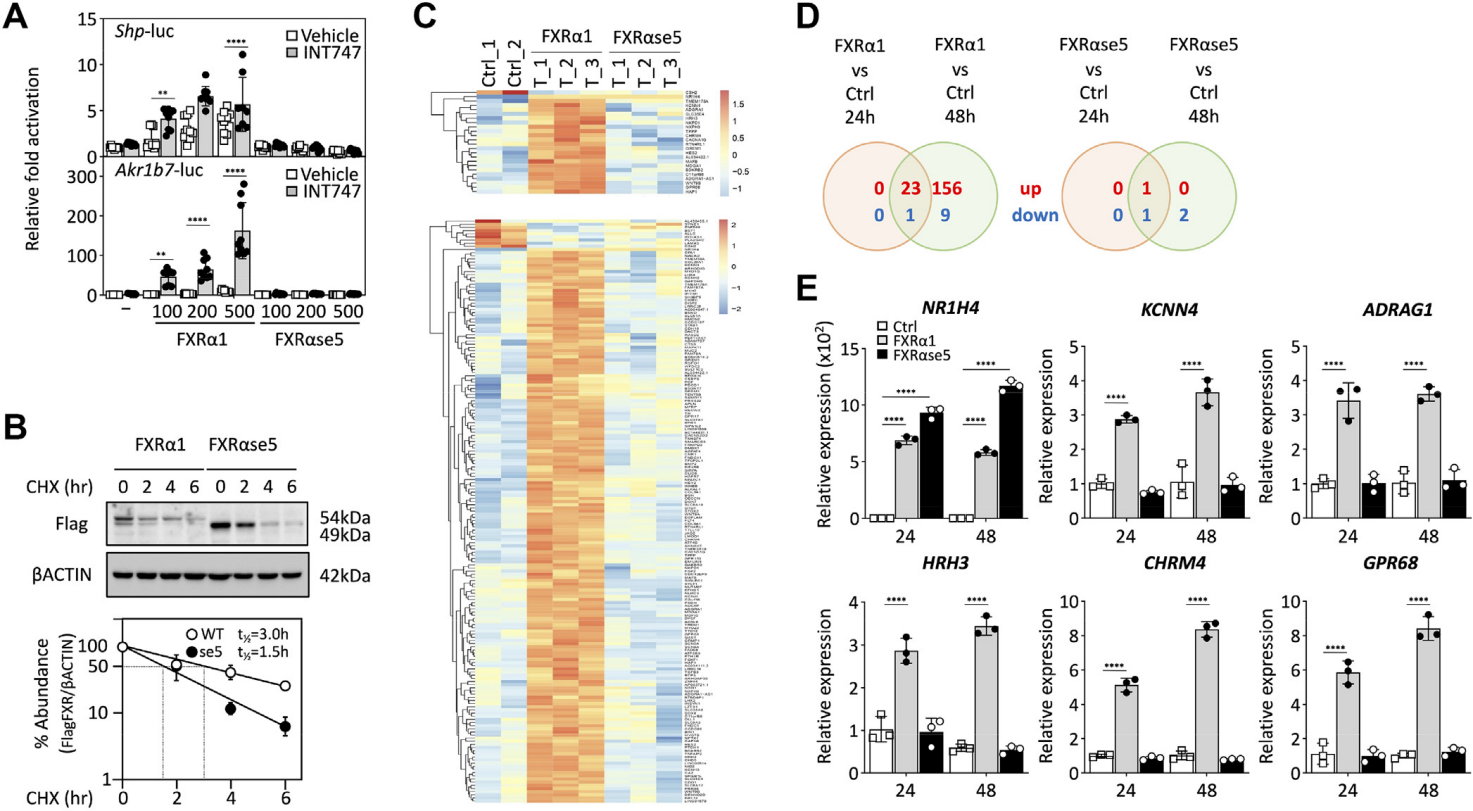
**Effect of alternative exon 5 splicing on the transcriptional activation of gene network by FXRα.***A*, HepG2 cells were cotransfected with the indicated reporter plasmids and various amounts (100–500 ng) of expression plasmids encoding for FXRα1 and exon 5 spliced FXRα variant as indicated. After 12 h, the cells were treated with 1 μM of INT-747 or vehicle (DMSO) and firefly luciferase activities were measured the following day. Luciferase activities were normalized relative to protein concentrations and results of the assay are expressed as fold-induction over empty vector (fixed to 1). The data were presented as mean ± SD (n = 9 biological replicates from three independent experiments). ∗*p* < 0.05, ∗∗*p* < 0.01, ∗∗∗*p* < 0.001, ∗∗∗∗*p* < 0.0001 *versus* nontransfected HepG2 cells. *B*, transfected HepG2 cells were treated with cycloheximide (CHX, 50 μM) for the indicated time period and Flag-FXRα levels in cell extracts were detected. *C*, Heatmap of gene expression profiles in HepG2 overexpressing FXRα1 and FXRαse5 spliced isoforms (*upper*, 24 h; *lower*, 48 h). *D*, Venn diagrams depicting the number of FXRα1-induced genes (*left*) and FXRαse5-modulated genes (*right*) compared to control nontransfected HepG2 cells 24 and 48 h after transfections. *E*, relative mRNA quantification (qPCR) of selected FXRα-modulated genes (time point 24 h in heatmap depicted in *C*) from independent cotransfection assays in HepG2 cells after 24 h and 48 h *versus* control cells (Ctrl). β-actin was used as a reference gene, and data are presented as mean ± SD from independent experiments (each symbol corresponds to one replicate). ∗*p* < 0.05, ∗∗*p* < 0.01, ∗∗∗*p* < 0.001, ∗∗∗∗*p* < 0.0001 *versus* nontransfected HepG2 cells. FXRα, farnesoid X receptor α; FXRαse5, spliced exon 5 FXRα; DMSO, dimethylsulfoxide.

To evaluate the role of FXRαse5 isoform as a ligand-dependent and/or a ligand-independent transcription factor, we assayed its ability to transactivate luciferase reporter genes driven by the IR-1 FXRE element of the small heterodimer partner (*Shp*) and aldo-keto reductase 1B7 (*Akr1b7*) promoters. HepG2 cells were transfected with various amounts of FXRαse5 encoding plasmid or WT FXRα1-encoding plasmid as this isoform possesses the highest transcriptional activity among all FXRα isoforms according to prior research (10, 11, 12). As depicted in Fig. 3*A*, increasing amounts of FXRα1 (100–500 ng) stimulate transcription of both *Shp* and *Akr1b7* promoter activities in HepG2 cells and the presence of the selective INT-747 agonist (1 μM for 24 h) further enhanced these two different FXRE-driven luciferase gene reporters. In contrast, FXRαse5 expressing plasmid failed to activate luciferase FXRE driven reporter genes even at high doses of INT-747 indicating that the splicing variant was unable to stimulate transcription of an FXRα target gene (Fig. 3*A*). Quantification of protein decay rate using cycloheximide treatment upon transient transfection in HepG2 revealed that the exon 5 spliced FXRα variant was slightly less stable than WT FXRα with half-lives of 1.5 h and 3.0 h, respectively (Fig. 3*B*). Although FXRαse5 isoform appeared to be more labile, the lack of transcriptional activation by this shorter isoform alternatively spliced for exon 5 may result from a reduced stability and/or difference in expression levels. To assess the impact of the FXRαse5 isoform in modulating FXRα transcriptional network through specific binding to other FXRE DNA sequence motifs, we used RNA sequencing to explore the transcriptome of HepG2 overexpressing either FXRα1 or FXRαse5. Comparing genes regulated by these two FXRα isoforms with the empty control vector, we generated a heatmap and Venn diagram of the RNAseq datasets (Fig. 3, *C* and *D*). The results indicate that FXRα1 isoform regulated a large gene set in comparison with the FXRαse5 isoform. However, none of the 179 genes upregulated by FXRα1 at 48 h are modulated by this isoform alternatively spliced for the second half of the DBD. Experimental validation of the gene expression data by reverse transcription-quantitative polymerase chain reaction (Fig. 3*E*) clearly revealed that FXRαse5 isoform was unable to recapitulate the gene programs activated by FXRα1; this finding suggests a contrasting effect owing to the loss of a key structural motif in the DBD with an impact on FXRα-regulated signaling pathway. These results raised questions regarding the ability of this variant in interacting with its heterodimer partner RXRα and binding efficiently to specific DNA motifs; this issue has been addressed below.

### Loss of exon 5 modifies RXR**α** interaction and impairs DNA binding of FXR**α**/RXR**α** heterodimers

The DBD of FXRα isoforms contains two highly conserved zinc finger motifs that form right-angle oriented helix ensuring contact with the major groove of the DNA and receptor dimerization with RXRα through molecular interactions that are not completely clear (Fig. 1*E*, inset). Given the importance of heterodimerization with RXRα in FXRα-driven transcriptional network, we investigated whether the loss of the second zinc finger of the DBD may affect the transcriptional activity of the FXRα/RXRα heterodimer as well as protein-protein and/or protein-DNA interactions required for cooperative dimeric binding of FXRα with RXRα to specific FXRE motifs.

**Figure 4:**
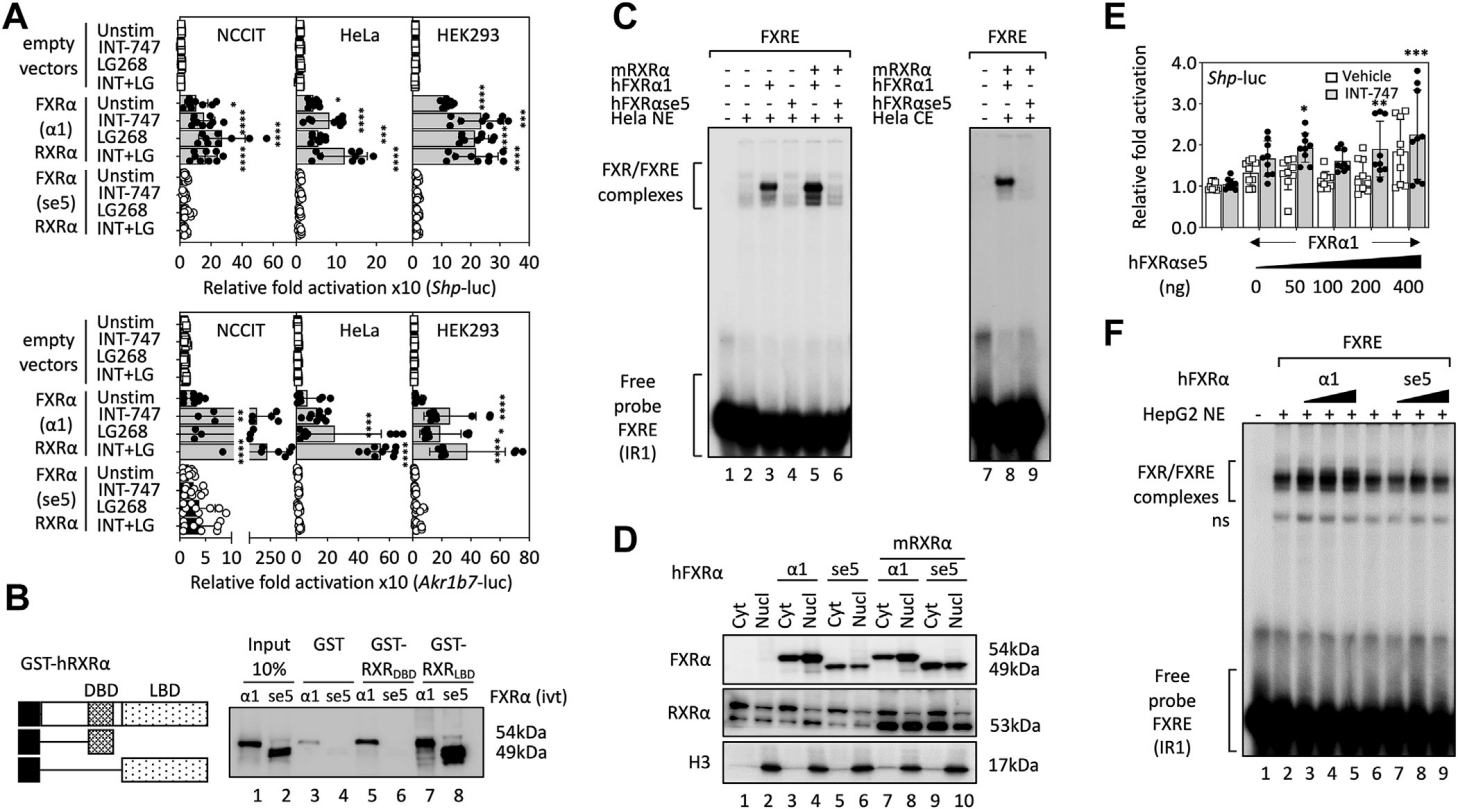
**Exon 5 splicing impairs FXRα transactivation and DNA-binding properties.***A*, cotransfected cells (NCCIT, HeLa, and HEK293) with full-length FXRα1 or exon 5-skipped isoforms expression plasmid, RXRα expression plasmid, and FXRE-responsive luciferase reporter genes. Cells were treated with vehicle (DMSO, unstimulated), 1 μM INT-747 (INT) and/or 1 μM LG100268 (LG268) for 24 h. Luciferase activity is shown normalized to vehicle-treated cells (fixed to 1) and was performed in triplicate, plotted with the mean ± SD and representative of independent experiments (n = 9; ∗*p* < 0.05, ∗∗*p* < 0.01, ∗∗∗*p* < 0.001, ∗∗∗∗*p* < 0.0001 compared to unstimulated empty vectors transfected cells). *B*, GST alone, or fused to the DBD or LBD of human RXRα were expressed in *Escherichia coli* and then purified with glutathione-agarose beads. Fusion protein-bound beads were incubated with *in vitro* translated FXRα isoforms, washed and then separated by 10% SDS-PAGE. Bound-FXRα isoforms were visualized by Western blotting using an anti-FXRα antibody. *C*, electrophoretic mobility-shift assay performed with the consensus IR1 FXRE and nuclear extract (NE) from HeLa cells overexpressing either FXRα1 or FXRαse5 individually or along with mouse RXRα, as indicated. *D*, nuclear (Nucl) and cytosolic (Cyt) fractions used in *C* analyzed using Western blotting with the indicated antibodies. Histone H3 detection was used to confirm the NE fractions. *E*, HepG2 cells were cotransfected with the FXRα1 plasmid and the *Shp*-luciferase reporter gene alone or with increasing ratio (0.5 to 4x) of FXRαse5 expression vector. Cells were incubated for 24 h with DMSO (vehicle) or with 1 μM INT-747. Luciferase activities were measured as described. Data are presented as mean ± SD of three independent experiments (n = 9; ∗*p* < 0.05, ∗∗*p* < 0.01, ∗∗∗*p* < 0.001, ∗∗∗∗*p* < 0.0001 compared to empty vector transfected cells treated with vehicle). *F*, increasing amounts of FXRα1 or FXRαse5 expressing vectors were transfected in HepG2 cells and NEs were incubated with a radiolabeled probe containing the consensus IR1 FXRE. Complexes were detected using gel mobility assay and nonspecific complex formed between the FXRE and HepG2 NE, as indicated (ns). DBD, DNA-binding domain; DMSO, dimethylsulfoxide; FXRα, farnesoid X receptor α; FXRαse5, spliced exon 5 FXRα; FXRE, FXRα response element; LBD, ligand-binding domain; RXRα, retinoid X receptor α; Shp, small heterodimer partner.

Using a series of transient transfection assays, we revealed that the RXRα-specific agonist LG100268 (LG268) stimulated transactivation of both *Shp*-, and *Akr1b7*-driven luciferase reporter genes by FXRα1/RXRα in three different cell lines (NCCIT, HeLa, and HEK293) and produced either additive or synergistic effects with the FXRα agonist INT-747 (Fig. 4*A*). However, overexpression of the FXRαse5 isoform silenced the integration of signals by RXRα and led us to hypothesize that the loss of the second zinc-finger rendered FXRα nonpermissive to RXRα heterodimer since the signal transmission by the two ligand-dependent signaling pathways was abrogated in the FXRαse5/RXRα heterodimer (Fig. 4*A*). The structural mechanisms affecting signal transmission by RXRα and the heterodimer partner FXRαse5 are still unknown; however, we were unable to exclude that loss of the second zinc finger of FXRα may promote allosteric modifications which may modify interactions with RXRα LBD, leading to conformational changes of the dimer interface and disruption of the adjacent coregulator- and/or ligand-binding sites, for silencing RXRα permissive heterodimers (15).

Therefore, we generated GST fusion proteins with the DBD and LBD domains of RXRα and used *in vitro*-synthesized FXRα to measure the binding capacity of the FXRαse5 isoform. As depicted in Fig. 4*B*, FXRα1 strongly associated with both the DBD and LBD domains of RXRα (compare lane 3 with 5 and 7) whereas the interaction of the FXRαse5 isoform was abrogated for the DBD domain of RXRα (compare lanes 5 and 6). On the contrary, the binding affinity of this isoform for the LBD domain of RXRα was markedly enhanced under the same condition (Fig. 4*B*, compare lanes 3 and 4 with lanes 7 and 8). Since dimerization with RXRα induces FXRα allosteric conformational changes and enhances its transcriptional activity (15, 16, 17), we further studied the interaction of the alternatively spliced FXRαse5 isoform for its DNA binding site using electrophoretic mobility shift assay (EMSA). Expression vectors for both isoforms were transfected individually (or along with RXRα) in HeLa cells and nuclear extracts (NEs) were prepared and incubated with a ^32^P-labeled double-stranded IR-1 consensus FXRE oligonucleotide for FXRα/RXRα DNA binding assessment. Specific FXRα-FXRE complexes were formed when WT FXRα1 was present in the NEs (Fig. 4*C*, compare lanes 2 and 3) and as expected, the presence of RXRα increased the intensity of the band containing specific FXRα-FXRE complexes (Fig. 4*C*, compare lanes 3 and 5). No specific complexes were detected with the FXRαse5 spliced isoform in the presence or absence of RXRα (lanes 4 and 6); moreover, cytoplasmic cell extracts as a source of protein also failed to identify sequence-specific binding for FXRαse5 (lanes 7–9) although comparable protein levels were detected in nuclear (Nucl) and cytoplasmic (Cyt) extracts using Western blot analysis (Fig. 4*D*, compare lanes 1, 2, and lanes 3–10). These results imply that deletion of the second zinc finger interferes with the capacity of FXRα to heterodimerize with RXRα and bind to a consensus IR-1 motif.

To further analyze the transcriptional properties of this alternatively spliced FXRαse5 isoform, we cotransfected FXRE-containing luciferase reporter genes with increasing amounts of the spliced isoform plasmid in HepG2 cells. Results indicate that increasing ratio of FXRαse5 isoform to WT receptor (from 0.5 to 4x) did not prevent transcription activation of a SHP-luciferase reporter gene (Fig. 4*E*). A gel mobility shift assay was performed to investigate whether this reduction is exerted by coregulators tethering and/or at the level of DNA binding. In this assay, NEs from HepG2 cells overexpressing either FXRα1 or its spliced exon 5 isoform were incubated with a ^32^P-labeled FXRE consensus probe. As expected, NEs of nontransfected HepG2 cells formed specific FXRα-FXRE complexes, which were enhanced with increasing amounts of transfected FXRα1 in HepG2 cells (Fig. 4*F*, lanes 2–5) but not with FXRαse5; this activity did not affect the intensity of the complexes (lanes 6–9) although FXRαse5 by itself was unable to bind to the FXRE (Fig. 4*C*). Overall, the FXRαse5 isoform not likely act as a weak repressor but it remains unclear if it can interfere with additional ligand-dependent signals integrated by other NRs. It is also unclear if the FXRαse5 isoform can interfere with the permissive dimer partner RXRα across the dimer interface since the isoform strongly interacts with the LBD of RXRα that rheostats RXRα permissiveness by conformational dynamic changes (18).

### Exon 5-skipping leads to FXR loss-of-function in hepatocarcinoma cells

**Figure 5:**
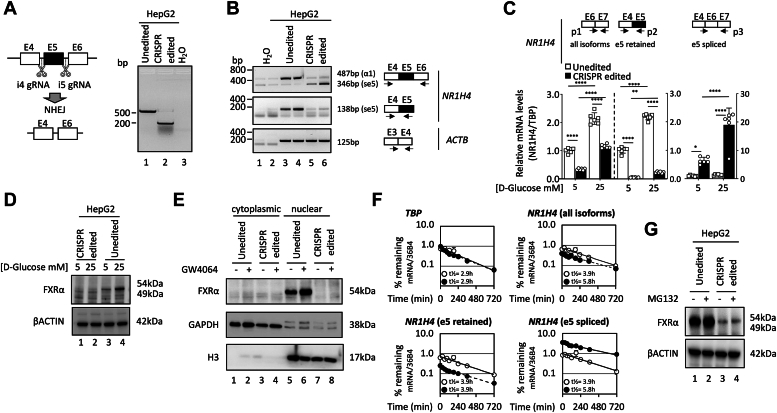
**CRISPR/Cas9-mediated genomic and transcript deletion of *NR1H4* exon 5.***A*, the Cas9 nuclease is targeted to introns 4 and 5 by two gRNAs (*left* panel). Simultaneous generation of double-stranded breaks (DSBs) by Cas9 leads to excision of the region surrounding the *NR1H4* exon 5. The distal ends are repaired through nonhomologous end joining (NHEJ). PCR across the genomic deletion region shows the smaller deletion PCR product in HepG2 CRISPR-edited cells (*right* panel). *B*, RT-PCR amplicons using primers (labeled with *arrows*) at specific exons were separated by 2% agarose gel electrophoresis; bands are marked based on the predicted sizes of the alternatively spliced variant shown at the side of each gel. *ACTB* was used as a reference gene. *C*, relative mRNA quantification of FXRα mRNA using RT-qPCR in CRISPR-edited cells with primer pair 3 (p3) spanning exons 4 and 6 depicting exon 5-skipping (*right*). RT-qPCR targeting conserved FXRα exons 6-7 junctions (primer pair 1, *left*) or exons 4 and 5 (primer pair 2, *middle*) were used as control for CRISPR cell editing. *TBP* was used as reference gene. Data are presented as mean ± SD of two independent experiments (n = 6; ∗*p* < 0.05, ∗∗*p* < 0.01, ∗∗∗*p* < 0.001, ∗∗∗∗*p* < 0.0001). *D* and *E*, Western blot analysis of FXRα. Cells were either cultured in low (5 mM) or high (25 mM) glucose media or with vehicle (DMSO) and GW4064 (1 μM) for 48 h, as indicated. Full-length FXRα1 is ∼54 kDa. FXRα without exon 5 (se5) is ∼49 kDa. β-ACTIN, GAPDH, and histone H3 serve respectively, as loading and cytosolic/nuclear protein fractionation controls. *F*, mRNA decay assays for WT FXRα1 (e5 retained) and exon 5-skipped (e5 spliced) isoforms in unedited (○) and CRISPR-edited (•) HepG2 cells. After 16 h of culturing in high glucose medium (25 mM), cells were incubated in low glucose medium (5 mM) and samples were collected at 0, 240, 480, and 720 min after addition of 5 μg/ml of actinomycin D. To compare mRNA decay curves, FXRα mRNA levels were expressed relative to 36B4 and the corresponding t = 0 point was set at 1. Results are representative of two independent experiments. *G*, Western blot analysis of FXRα isoform abundance in control and CRISPR-engineered HepG2 cells treated with vehicle (DMSO) or 40 μM MG132 for 12 h. FXRα, farnesoid X receptor α; DMSO, dimethylsulfoxide; RT-qPCR, reverse transcription-quantitative polymerase chain reaction.

Variations in the proportions of *NR1H4* exon 5-skipping in different cell lines and tissues of human origin (Figs. 1*G* and 2) raised questions with regard to the biological relevance of this splicing event. Our data clearly indicates that the FXRαse5 isoform reveals no detectable binding to a consensus FXRα/RXRα-binding site or transcriptional activity in response to the synthetic agonist INT-747 on IR-1 elements from the *Shp* and *Akr1b7* promoters (Figs. 3 and 4). Moreover, there is no obvious mechanism for this FXRαse5 variant to exert a dominant negative effect contrarily to other alternatively spliced NRs described so far (19, 20). To further explore the functions of this minor isoform, compared to the predominant FXRα RNA transcript (Fig. 2, *B* and *C*), we genome-edited *NR1H4* exon 5′ and 3′ boundary regions to induce nonhomologous end-joining leading to in frame exon 5-skipping. As depicted in Fig. 5*A*, the dual guide RNA approach designed to remove a 381 base pair fragment promoted exon 5-skipping (compare lanes 1 and 2). Homozygous deletion was confirmed using genomic DNA-PCR analysis and Sanger sequencing. As presented in Figure 5, *B* and *C*, the genetic outcome of CRISPR-directed gene editing was mapped at the transcriptional level. Total RNA isolated and analyzed by RT-PCR using exons 4 and 6 targeting primers (labeled by arrows) revealed two transcript populations after gel electrophoresis separation reflecting inclusion or excision of exon 5 (Fig. 5*B*, upper panel). Detection of a specific band only in the unedited HepG2 cell population using primers that targeted exons 4 and 5 reflected the proper editing of the *NR1H4* gene, for exon 5-skipping based on the predictive size of 138 bp (middle panel, lanes 3–4 compared with 5–6). Relative mRNA quantification of FXRα isoforms was selectively obtained using primer pair annealing on specific exons and/or at exon-exon junctions as schematized in Fig. 5*C* (top panel). Notably, total FXRα transcripts (all isoforms) detected with primers on exons 6 and 7 (primer pair 1, p1) were reduced in CRISPR-edited cells by 60 and 50%, respectively, in low (1.00 ± 0.09 *versus* 0.34 ± 0.03, *p* < 0.0001) and high (2.14 ± 0.20 *versus* 1.15 ± 0.07, *p* < 0.0001) glucose conditions, when compared to the HepG2 unedited control cell line. Amplification of FXRα variant retaining exon 5 (e5 retained) in unedited HepG2 cells was obtained by primer pair 2 (p2) targeting exons 4 and 5. Inclusion of exon 5 in low glucose cultured CRISPR-edited cells was decreased by 95% (0.05 ± 0.01 *versus* 1.01 ± 0.11, *p* < 0.0001) compared to control HepG2 indicative of exon 5-skipping in CRISPR engineered HepG2 cells. Incubation in high concentrations of D-glucose (25 mM) increased exon 5-retained FXRα mRNA by more than two-fold (2.17 ± 0.10 *versus* 0.24 ± 0.03, *p* < 0.001) after 48 h in unedited HepG2 compared to low D-glucose condition (5 mM). Analysis using a forward primer spanning the junction between exons 4 and 6 with a reverse primer in exon 7 (primer pair 3, p3) also displayed exon 5-skipping with a marked increase of exon 5-spliced FXRα mRNA in response to glucose (25 mM) in HepG2 engineered cells. These results indicate that regulation of FXRαse5 mRNA level is influenced by glucose and suggests that glucose-regulated FXRα gene expression mechanisms, described previously, are conserved for this variant isoform (21). As FXRαse5 variant lacking a functional DBD does not appear to exhibit dominant-negative functions, we examined whether an alternative exon 5 excision in the FXRα could create a loss-of-function phenotype.

Western blot revealed that WT FXRα was expressed and detected at 54 kDa in unedited HepG2 cells while a faint but visible band under 50 kDa was observed after CRISPR alterations of *NR1H4* gene reflecting the exon 5-skipped transcript detected with a 47 amino acid deletion corresponding to a 141 base pair excision (Fig. 5*D*, lanes 3 and 4 compared with lanes 1 and 2). Incubation in high concentrations of glucose (25 mM) resulted in an increase of FXRα protein abundance after 48 h in unedited parental HepG2 (compare lanes 3 and 4) but not in CRISPR-edited cells (compare lines 1 and 2). Moreover, the FXRα agonist GW4064 failed to increase the nuclear accumulation of the alternatively spliced FXRα variant lacking exon 5 (Fig. 5*E*, compare lanes 5–6 to 7–8). RNA interference-mediated protein knockdown validated the FXRα antibody value for Western blotting application (Fig. S2). To examine whether FXRαse5 reduction of mRNA and protein accumulation resulted from lower stability, experiments were performed using the RNA polymerase inhibitor actinomycin D and the MG132 proteasome inhibitor. Cells were preincubated overnight in a high concentration of D-glucose (25 mM) to increase mRNA levels. The medium was then changed and cells were incubated with actinomycin D in the presence of low D-glucose concentration for mRNA evaluation at different time points (Fig. 5*F*). Comparison of mRNA decay curves revealed that unspliced FXRα isoform (exon 5 retained) mRNA half-life was identical in both unedited (white circle) and CRISPR-edited (black circle) cell lines; however, the steady-state mRNA levels for FXRα exon 5 spliced isoforms (exon 5 spliced) were slightly higher in CRISPR-edited (black circle) cells harboring FXRα loss-of-function. As our global analysis of FXRα isoforms did not reveal marked changes in mRNA half-lives of both isoforms, we examined the role of the MG132 proteasomal inhibitor to determine whether the ubiquitin-proteasome system shortened FXRαse5 protein half-life posttranslation. Inhibition of the proteasome did not increase exon 5-spliced FXRα isoform protein level (Fig. 5*G*) indicating that increased protein degradation was probably not the dominant mechanism leading to the suppression of FXRαse5 protein accumulation. To reconcile these differences, we hypothesized that FXRα exon 5-spliced mRNA transcripts may be translated at a very low level and possibly not completely depleted by a nonsense-mediated RNA decay pathway.

### Splicing inhibition of FXR**α** changes cell metabolism in conjunction with LRH-1 and CD36 upregulation

**Figure 6:**
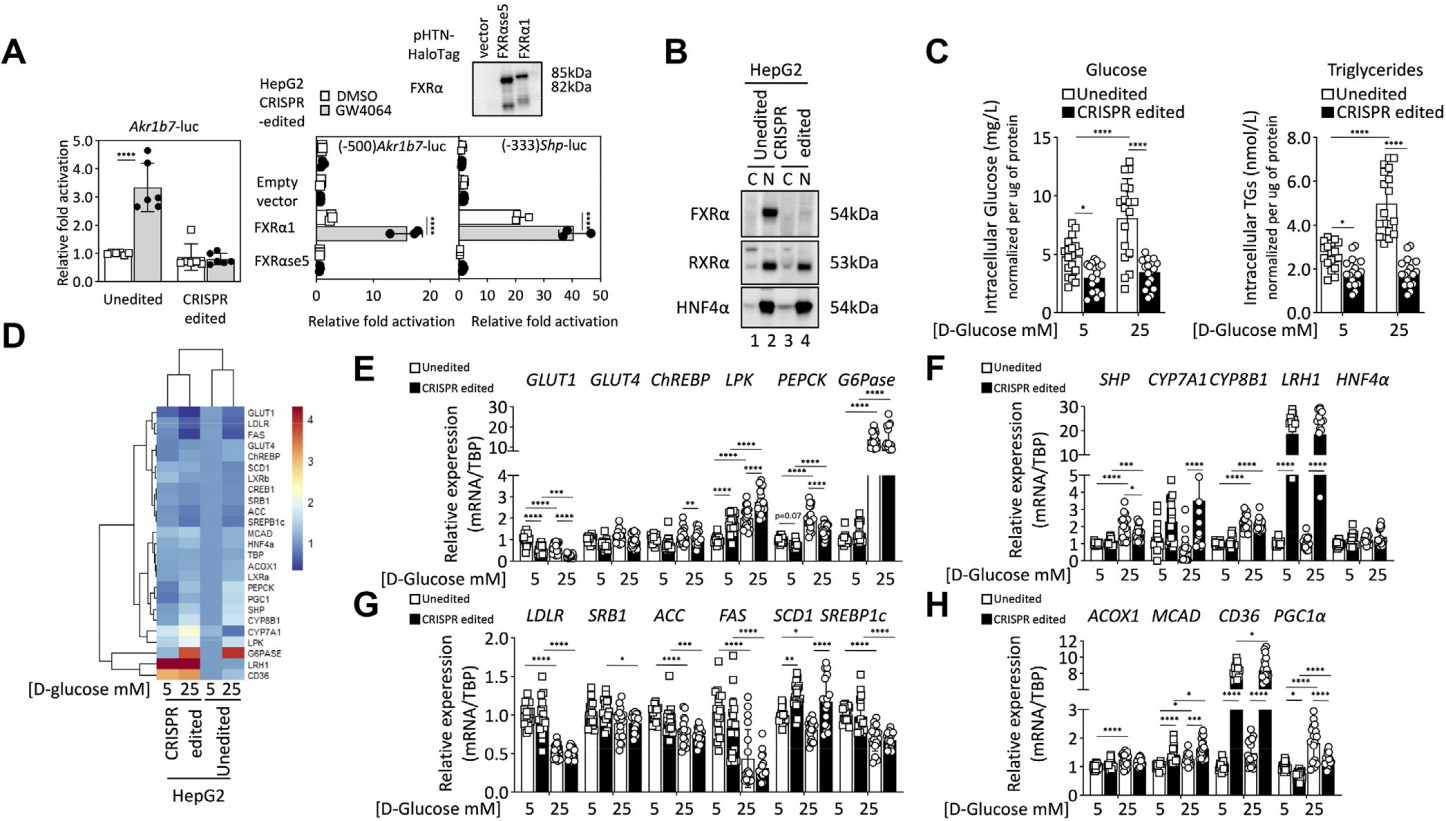
**Alternative splicing of exon 5 impairs FXRα function and reshapes target genes balancing energy and nutrient metabolism.***A*, exon 5-skipping leads to FXRα loss-of-function. Unedited and CRISPR-edited HepG2 cells with skipped exon 5 were transiently transfected with the *Akr1b7*-luciferase reporter gene in the presence or absence of the FXRα agonist GW4064 (1 μM). After 48 h, cells were harvested and lysed for luciferase activity assessment (*left*). Data are presented as mean ± SD of two independent experiments (n = 6; ∗*p* < 0.05, ∗∗*p* < 0.01, ∗∗∗*p* < 0.001, and ∗∗∗∗*p* < 0.0001 compared to unedited DMSO treated cells). Rescue of FXRα transcriptional activity by transient transfections of FXRα1 and FXRαse5 Halo-tag expression vectors in genome-edited HepG2 cells. *Akr1b7* and *Shp*-driven luciferase activities were measured after 48 h in the presence of DMSO as vehicle or 1 μM GW4064 (*right*). Results are presented as mean ± SD (n = 3 for each reporter gene). Insert shows expression levels of recombinant Halo-tagged FXRα proteins. *B*, induced FXRα loss-of-function by CRISPR/Cas9-mediated genome editing of exon 5 does not alter abundance of master regulators of hepatic differentiation including HNF4α. Cytosolic and nuclear fractions were analyzed using Western blotting with the indicated antibodies. *C*, FXRα impairment by exon 5 splicing modulates glucose and triglyceride homeostasis. Cells were serum-starved overnight in low glucose containing medium, before incubation with low or high glucose media supplemented with 10% FBS. Cells were lysed 48 h later and both intracellular glucose and triglycerides were evaluated. Data are presented as mean ± SD of three independent experiments (n = 18; ∗*p* < 0.05, ∗∗*p* < 0.01, ∗∗∗*p* < 0.001, ∗∗∗∗*p* < 0.0001 versus control low glucose cultured HepG2 unedited cells). *D*, Z-score hierarchical clustering and heat map visualization of gene expression profiles involved in glycolytic and lipogenic metabolisms, bile acid homeostasis, and fatty acid uptake and oxidation (*E*–*H*). Colors represent scaled expression values, with *blue* for low and *red* for high expression levels. Quantification of gene expression by RT-qPCR is presented as mean ± SD of three independent experiments (n = 18; ∗*p* < 0.05, ∗∗*p* < 0.01, ∗∗∗*p* < 0.001, ∗∗∗∗*p* < 0.0001 *versus* control low glucose cultured HepG2 unedited cells). Akr1b7, aldo-keto reductase 1B7; DMSO, dimethylsulfoxide; FBS, fetal bovine serum; FXRα, farnesoid X receptor α; FXRαse5, spliced exon 5 FXRα; HNF4, hepatocyte nuclear factor 4; RT-qPCR, reverse transcription-quantitative polymerase chain reaction; Shp, small heterodimer partner.

To confirm the FXRα loss-of-function phenotype induced by CRISPR/Cas9-mediated genome editing of exon 5, cells were transiently transfected with the *Akr1b7*-luciferase reporter. As depicted in Fig. 6*A* (left), luciferase activity was not induced in CRISPR-edited cells 48 h post transfection regardless of the presence or absence of the FXRα agonist, GW4064 (1 μM). On the contrary, luciferase activity was stimulated greater than 3-fold (3.33 ± 0.86 *versus* 1.00 ± 0.04, *p* < 0.0001) in unedited HepG2. Off target effects in CRISPR/Cas9 gene editing cannot explain the absence of luciferase activity in cells edited for *NR1H4* exon 5 excision since transient transfection of an expressing vector for WT FXRα rescued both *Shp*- and *Akr1b7*-driven luciferase activity (Fig. 6*A*, right). Moreover, expression of hepatocyte nuclear factor 4 α (HNF4α), which acts as the main regulator of hepatic differentiation (22), suggests that genome edited hepatocytes maintained their state of differentiation and ability to regulate nutrient metabolism (Fig. 6*B*).

To evaluate whether splicing inhibition of FXRα represented a regulatory mechanism to control energy balance, intracellular glucose, and triglyceride contents were measured. Cells were preincubated overnight in serum-free Dulbecco’s modified Eagle medium (DMEM) with low concentration of D-glucose (5 mM). The following day, the medium was changed, and cells were incubated for an additional 48 h in low or high glucose containing medium with 10% fetal bovine serum (FBS) as depicted in Fig. 6*C*. Media were refreshed after 24 h to avoid nutrient depletion. CRISPR-edited cells cultured in low D-glucose medium displayed lower intracellular glucose and triglyceride levels compared to the unedited HepG2 parental cell line (glucose: 3.01 ± 1.23 mg/L *versus* 4.82 ± 1.59 mg/L, *p* < 0.05; triglycerides: 1.82 ± 0.62 nmol *versus* 2.62 ± 0.59 nmol, *p* < 0.05). When cultured in high glucose medium, intracellular glucose and triglyceride contents were doubled in HepG2 unedited cells (glucose: 8.10 ± 3.37 mg/L *versus* 4.82 ± 1.59 mg/L, *p* < 0.0001; triglycerides: 4.98 ± 1.42 nmol *versus* 2.62 ± 0.59 nmol, *p* < 0.0001) however, no difference was observed in exon 5-skipped genome-edited cells (glucose: 3.48 ± 1.20 mg/L *versus* 3.01 ± 1.23 mg/L, *p* = 0.899; triglycerides: 1.82 ± 0.62 nmol *versus* 1.82 ± 0.62 nmol; *p* = 0.999). No significant difference was observed after quantification for residual glucose percentage in low (1.0 g/L) and high (4.5 g/L) glucose-containing media after 24 h (low: unedited 42.7% ± 0.34 *versus* CRISPR 42.67% ± 0.34; high: unedited 86.70% ± 1.39 *versus* CRISPR 83.37% ± 1.21). These results indicate that exon 5-skipping by alternative splicing of the *NR1H4* gene suppressed FXRα signaling with an impact on both glucose and lipid homeostasis.

To determine whether the reduction in glucose and triglyceride intracellular accumulation translated into variations in FXRα target genes balancing energy and nutrient metabolism, we evaluated the transcriptional network changes and profiled global gene expression in cell lines expressing the WT FXRα or FXRαse5 variant in low and high glucose conditions. By comparing differentially regulated gene sets, we generated a heatmap (Fig. 6*D*) depicting reverse transcription-quantitative polymerase chain reaction values for metabolic genes controlling lipid and glucose homeostasis (Fig. 6, *E*–*H*). FXRα loss-of-function by exon 5-skipping increased *CYP7A1* expression according to the well-described function of this NR in the regulation of bile acid homeostasis (23). Notably, FXRα suppression robustly induced liver receptor homolog LRH-1 transcripts (encoded by *NR5A2*) by over 18-fold (low: 18.69 ± 9.67 *versus* unedited control: 1.01 ± 0.17, *p* < 0.0001; high: 18.56 ± 10.37 *versus* unedited control: 1.05 ± 0.32, *p* < 0.0001), independently of glucose condition (Fig. 6*F*). LRH-1 is described as a key regulatory component of the hepatic glucose-sensing system required for integration of glucose and lipid metabolism (24). Decrease of phosphoenolpyruvate carboxykinase (PEPCK; low: unedited control 1.08 ± 0.13 *versus* CRISPR edited: 0.75 ± 0.14, *p* = 0.075; high: unedited control 2.02 ± 0.53 *versus* CRISPR edited: 1.47 ± 0.30, *p* < 0.0001) mRNA together with the increase in L-type pyruvate kinase (low: unedited control 1.02 ± 0.20 *versus* CRISPR edited: 1.76 ± 0.45, *p* < 0.0001; high: unedited control 2.01 ± 0.51 *versus* CRISPR edited: 2.76 ± 0.58, *p* < 0.0001) expression (Fig. 6*E*) suggests that the glycolytic rate may be higher than gluconeogenesis in CRISPR-edited cells. Moreover, reduced expression of both lipogenic genes acetyl-coenzyme A carboxylase and fatty acid synthase indicated that endogenous fatty acid synthesis may also be impaired when FXRα was inactivated by splicing without changes in expression of low-density lipoprotein receptor and scavenger receptor B1 (SRB1) lipoprotein receptors (Fig. 6*G*). Notably, expression of fatty acid translocase CD36 was elevated in the absence of FXRα whereas both glucose transporters (GLUT1, 4) were reduced (Fig. 6, *E* and *H*). This finding may reflect the need to fuel the cell by increasing fatty acid uptake when glucose availability was lower in order to sustain fatty acid oxidation and mitochondrial breakdown into acetyl-CoA units for ATP production. Moreover, the medium-chain acyl-CoA dehydrogenase that catabolizes the first step of fatty acid oxidation in mitochondria was also upregulated with CD36 (Fig. 6*H*). Together, these data suggest that inhibition of FXRα function by alternative splicing of exon 5 may activate LRH-1 to adapt metabolism to energy resources.

**Figure 7:**
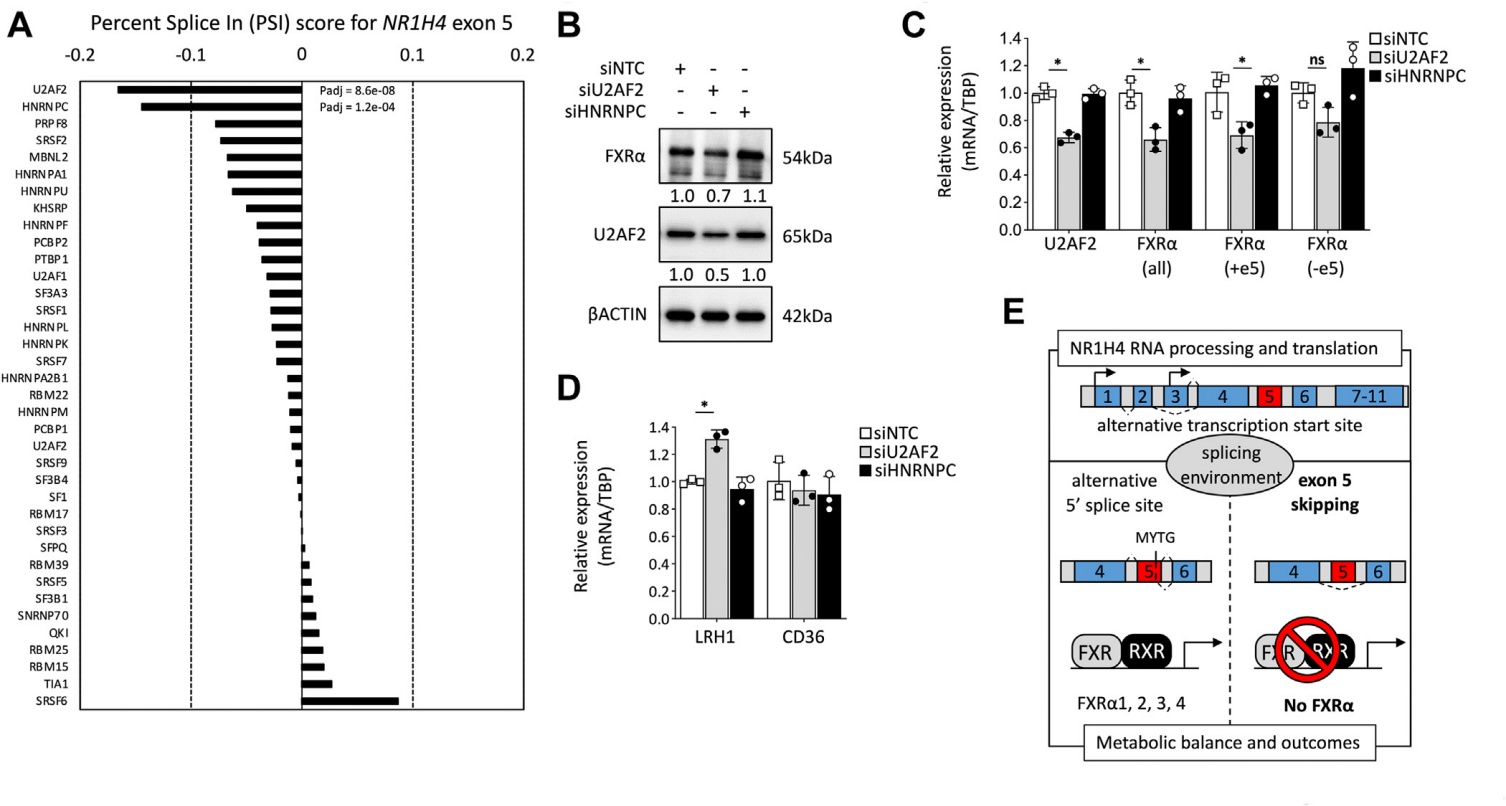
**Regulation of U2AF2 as a legitimate event for FXRα exon 5 splicing and adaptation of metabolic pathways.***A*, the Percent Splice In (PSI) score for exon 5 of *NR1H4* is presented for each splicing factor analyzed using the SplicingLore web resource database. *B* and *C*, Western blot and RT-qPCR analysis revealing knockdown of U2AF2 in human HepG2 hepatocarcinoma cells (n = 3) and expression of FXRα following treatment with siRNA targeted against nontargeting control, U2AF2, and hnRNPC. FXRα isoforms with exon 5 inclusion (+e5) or exclusion (−e5) were quantified using RT-qPCR with overall FXRα isoforms (all). Specific primers targeting exons, or exon-exon junctions are listed in Table 1. *D*, depletion of U2AF2 by siRNA increases LRH1 expression. Results are presented as mean ± SD from independent experiments (each symbol corresponds to one replicate). The *p*-values for ∗*p* < 0.05, ∗∗*p* < 0.01, ∗∗∗*p* < 0.001, ∗∗∗∗*p* < 0.0001 are shown in the corresponding images. *E*, schematic overview of alternative FXRα gene function with the expression of exon 5-skipped splice variant. The diversity of FXRα isoforms detected in human and mouse tissues depends on two alternative transcription start sites and on an alternative 5′ splice site. A change in the environmental conditions and/or abundance of splicing factors can shift the balance patterns toward the production of any of the FXRα transcript variants. Our research suggests that FXRα-expressing cells also contain a proportion of FXRα mRNAs derived from a DNA-binding domain (DBD) alternative splicing event leading to a loss-of-function isoform. This finding may represent a novel mechanism of FXRα alternative splicing regulation to adapt metabolic outcomes in response to environmental conditions and/or early onset of disease. FXRα, farnesoid X receptor α; hnRNPC, heterogeneous nuclear ribonucleoprotein C; LRH1, liver receptor homologue 1; RT-qPCR, reverse transcription-quantitative polymerase chain reaction; U2AF2, U2 small nuclear auxiliary factor 2.

Using the SplicingLore web resource, we were able to retrieve the most susceptible splicing factors (SFs) controlling the exclusion of exon 5 (exon 7 in FasterDB database) in the *NR1H4* gene product (25). Among the top predicted factors, the U2 small nuclear auxiliary factor 2 (U2AF2) and the heterogeneous nuclear ribonucleoprotein C (hnRNPC) were the best correlated factors targeting *NR1H4* exon 5 with respective change in percent spliced index (ΔPSI) values of −0.166 (*p* value = 8.62e^−8^) and −0.144 (*p* value = 1.17e^−4^), respectively, based on available RNA-seq datasets of KO cells for genes encoding SF proteins (Fig. 7*A*). Notably, siRNA-mediated U2AF2 silencing in HepG2 cells (−33% *versus* control siNTC; 1.00 ± 0.05 *versus* 0.68 ± 0.04, *p* < 0.0001) significantly reduced the abundance of FXRα protein by approximately 30% than a nontargeting control (NTC) siRNA (Fig. 7*B*). This decrease is associated with a 30% downregulation of global FXRα transcripts (0.66 ± 0.04 *versus* 1.00 ± 0.09; *p* = 0.01) as well as of the exon 5-retained isoform (0.69 ± 0.10 *versus* 1.01 ± 0.15; *p* < 0.05), and to a lesser extent, to the reduction of exon 5-spliced FXRα isoform (0.79 ± 1.09 *versus* 1.00 ± 0.07; *p* = 0,31) compared to NTC siRNA (Fig. 7*C*). The latter result was unexpected because we expected an increase in *NR1H4* mRNA levels with exon 5-skipping. However, the remaining WT FXRα buffers mRNA stability by regulating RNA binding proteins and/or *cis*-acting elements to control splicing events and thus regulates the mRNA half-life in liver cells during liver disease and hepatocellular carcinoma (HCC) (26). This action may explain the longer half-life observed for exon 5-skipped FXRα isoform in CRISPR edited cells from 3.8 to 5.9 h (Fig. 5*F*). Finally, the modest but significant increase in LRH-1 mRNA levels after U2AF2 silencing (1.32 ± 0.07 *versus* 1.00 ± 0.02; *p* < 0.05) may have resulted from FXRα splicing as an early event that may have primed the transcriptional activation of the CD36 gene (Fig. 7*D*). Together, these results indicate that exon 5 FXRα splicing may coordinate an adaptative response to fine-tune lipid and glucose metabolism through an LRH-1 regulated transcriptional network, increasing fatty acid uptake by CD36 to ensure nutrient homeostasis and metabolic flexibility.

## Discussion

The NR FXRα acts as a ligand-inducible transcription factor that governs distinct biological processes through the modulation of target gene expression with its obligate RXRα heterodimer partner. Advances in structural characterization and modeling of the *NR1H4* gene have led to the description of four FXRα isoforms in humans and mice, as a result of differential use of promoters and alternative RNA splicing. Therefore, many FXRα target genes are reported to be regulated in an isoform-specific manner to different extents, according to an extended N terminus, which encompasses the defined activation function 1 domain, and the presence or absence of a four amino acid MYTG motif located at the 3′ end of the DBD. Thus, as for most multiexon genes, alternative splicing plays a pivotal role in triggering a diverse set of functions. The *NR1H4* also uses alternative splicing to transduce adaptive and physiological responses after binding of small lipophilic ligands serving as a transcriptional switch.

We demonstrate that alternative splicing generates a novel FXRα isoform identical to the human FXRα1 but lacking the entire exon 5 (e5) that encodes the second zinc finger of the DBD. We have described this FXRαse5 isoform in human cells and tissues; it also exists in male and female adult mouse tissues. Based on amino acid alignments (this study and (10)), it would be expected that this splicing event affects all FXRα isoforms in humans as well as FXRα RNA transcripts in species other than mice; however, these possibilities have not been validated yet. A recent study has reported a naturally occurring splice variant pattern for the human NR family using a cassette exon visualization pictograph methodology, for illustrating the location of modular, cassette exons, in all groups of NR genes (27). From this, the elaborate cassette exon signatures of the *NR1H4* gene highlight a large array of cassette exons that include exon 5 suggesting that further FXRα splice variants may be generated with different DNA-binding properties, heterodimer interactions in order to refine complex physiological responses along with coregulatory proteins (this study and (28)). Pre-mRNA splicing and alternative splicing are contributing mechanisms to control liver adaptation, physiology, and homeostasis. These complex mechanistic events involve multiple *cis* and *trans* elements to adapt responses to metabolic stimuli in physiological and pathological contexts (29). In an attempt to functionally characterize this minor FXRαse5 isoform, we demonstrated that the U2AF2 auxiliary factor may play a role in FXRα pre-mRNA splicing by promoting the inclusion or skipping of exon 5 along with RNA-binding proteins. However, hnRNPC single repression did not change FXRα mRNA and protein levels substantially; moreover, it cannot be excluded that other SF are required to impact exon 5 alternative splicing and experimental validation of the SplicingLore predictions is required. Functional interaction between U2AF2 and hnRNPC are currently under investigation to validate and evaluate the interaction of these splicing regulators in directing the spliceosome assembly to the 3′ splice site of the alternative cassette exon 5. This finding may shed light on the role of alternative splicing in normal liver physiology and dysregulation in liver diseases (Fig. 7*E*).

Increasing evidence shows that alternative splicing contributes to liver homeostasis in a growing number of physiological contexts. Nutrient availability influences temporally, splicing patterns in the liver to regulate both glucose and lipid metabolism (30, 31). Inhibition of FXRα functions by exclusion of *NR1H4* exon 5 may play a dynamic role in the liver; although the metabolic signals remain elusive, the fatty acid uptake by CD36 may be orchestrated by LRH-1 when nutrient supply is limited during prolonged fasting. Similar to many other genes involved in lipid metabolism, CD36 expression is upregulated by the nuclear hormone transcription factor peroxisome proliferator-activated receptor γ (PPARγ) (32), and it may be proposed that activated PPARγ ligand synthesis by LRH-1 (33) may enhance CD36 gene transcription to facilitate fatty acid uptake and transport into the mitochondrial matrix for oxidative phosphorylation to fuel the cell activation response. CD36 levels are higher in patients with nonalcoholic fatty liver disease (NAFLD), possibly leading to the development of nonalcoholic steatohepatitis (32). Moreover, elevated CD36-dependent free fatty acid uptake was associated with the activation of proinflammatory pathways and promotion of epithelial–mesenchymal transition in HCC (34). Notably, a number of transcriptional effectors including NF-κB are activated by free fatty acids and activation of a NF-κB driven luciferase reporter gene in FXRα loss-of-function CRISPR edited HepG2 cells support the relationship of exon 5 alternative splicing with activation of proinflammatory pathways in the liver (Fig. S3). This information warrants further investigation relative to the role of the FXRαse5 variant in early NAFLD through HCC as dysregulation of RNA splicing is linked to the trigger of liver diseases and pathogenesis of HCC (26). As a master regulator of hepatic nutrient metabolism, FXRα serves as a pharmacological target for liver disease and metabolic disorders including type 2 diabetes (T2D). Hepatic FXRα expression is observed to be reduced in patients with nonalcoholic steatohepatitis and structurally optimized FXRα agonists generate wide attention because of the highly promising FXRα targeting in NAFLD treatment (35). Along with other NRs, FXRα also plays a key role in T2D pathogenesis and appears to be a potential pharmaceutical target for the treatment of T2D and its complications (36). Inactivation of FXRα *via* alternative splicing of exon 5 needs to be explored further in liver physiology and pathology, with more detailed mechanistic views that include gene regulation through nonsense-mediated RNA decay as a potential pathway to prevent FXRαse5 protein accumulation.

Although expression of the variant FXRαse5 protein was minimally detected in CRISPR-edited hepatocarcinoma cells, we cannot exclude its expression at higher levels in other conditions. Structural analysis of steroid receptor DBDs have revealed that the second zinc finger allows the receptor to interact with its homodimeric or heterodimeric partner while the first zinc finger makes the receptor-DNA contact by binding to a specific hormone response element. As described for other DBD-spliced steroid NRs such as the estrogen receptor (37), the binding of exon 5-skipped FXRα isoform to its consensus FXRE response element in the presence or absence of its RXRα heterodimeric partner may be possible. This action may be explained in part by the loss of the D-box included at the N terminus of the second zinc finger (Fig. 1*E*) as this five-amino acid motif regulates intramolecular interactions, but not direct DNA-binding (38). Many NR variants with skipped DBD encoding exons have been associated with human diseases including cancer (27); however, the structural consequence of exon 5 FXRα splicing in human disease promotion and health resilience are currently difficult to predict. A recent study reporting FXRα loss-of-function variants in patients with progressive familial intrahepatic cholestasis characteristics (39) presumes that splice inhibition of FXRα may be associated with early cholestasis and rapid progressive hepatic dysfunction in humans.

In addition to alternative splicing, changes in PTMs also affect NR activities and their regulatory effects on FXRα accumulation under physiological and pathological conditions drawing attention to conserved phosphorylation and acetylation sites within the second zinc finger motif in the DBD. Human FXRα ubiquitination is regulated either by SUMOylation of lysine 325 (40), acetylation of lysine residues 157 and 217 (41), or phosphorylation of serine 154 (42); however, a proteasome-mediated degradation process not likely accounts for the poor expression of the FXRαse5 isoform because exon 5-skipping removes both serine 154 (S154) and lysine 157 (K157) limiting extensive PTMs of the DBD associated with the ubiquitin-proteasomal degradation of FXRα.

Finally, pull-down assays, luciferase reporters, and transcriptome sequencing of HepG2 cells overexpressing either FXRα1 or FXRαse5 isoforms reveal that exon 5-skipping deprives FXRα metabolic receptor of its binding and gene transcription activities. *In vitro* interaction assay reveals that FXRαse5 strongly interacts with the LBD of RXRα but not with the DBD, in contrast to WT FXRα1 that interacts equally with both RXRα DBD and LBD. A plausible explanation for these differential molecular interactions between FXRα variants and RXRα may reside in the unique feature of the DBD of FXRαse5. This DBD lacks the second zing finger motif that encompasses the D-box, and the carboxy terminal extension of the DBD making an unfavorable global folding pattern for the interaction with the DBD of RXRα. Recently, intramolecular interactions between the DBD and LBD of the constitutive androstane receptor (CAR) NR has been demonstrated to regulate its conversion between an inactive or active heterodimer with RXRα (43). Notably, this previous work also reported that deletion of the hinge region reduces the flexibility of the CAR NR to correctly position its DBD relative to the LBD, and abrogates its ability to form an active RXRα heterodimer. Based on these observations, we postulate that FXRα1 exon 5-skipping by alternative splicing may reduce separation between the DBD and LBD to hinder intramolecular interactions creating an inactive FXRα partner that silences the permissive RXRα heterodimer as noted from our luciferase reporter gene assays. To explore this way, an immunoprecipitation-based screening is ongoing to define FXRαse5 interactome, but coimmunoprecipitation using either Flag- or Halo-tagged FXRα recombinant proteins failed to precipitate known cofactors including p300, Sirt1, and RXRα. Hence, crystal structure and three-dimensional DNA modeling of FXRα1 will be of interest to better understand the structural consequence of exon 5-splicing on FXRα outputs within its permissive RXRα heterodimer. In summary, these findings establish alternative splicing as a complementary mechanism of FXRα signaling regulation, opening new prospects for the study of FXRα in physiological and pathologic conditions. Indeed, both high throughput screening for differential FXRαse5 cofactors and analysis of liver transcriptomic signatures from patient with liver diseases are now undergoing in our lab to shed insights in delineating molecular cues to elucidate how this new isoform regulates FXRα signaling function.

## Experimental procedures

### Materials and plasmid constructs

Gibco supplied DMEM containing 1.0 g/L glucose (21885025) or 4.5 g/L glucose (11960044), Roswell Park Memorial Institute medium (RPMI 1640, 318700874), Opti-MEM (31985062), and penicillin/streptomycin (15140122). FBS (CVFSVF00–01), L-glutamine (CSTGLU00-0U), and DPBS (CS1PBS01–01) were obtained from Eurobio Scientific. The proteasomal inhibitor MG132 (40 μM, Sigma-Aldrich) was added to the medium where needed. Antibodies used were mouse monoclonal anti-FXRα (A9033A, Perseus Proteomics); rabbit polyclonal anti-RXRα (sc-553, Santa Cruz Biotechnology); goat polyclonal anti-H3 (sc-86534, Santa Cruz Biotechnology); rabbit monoclonal anti-U2AF65 (ab197031, Abcam); rabbit polyclonal anti-HNF4α (3113S, Cell Signaling Technology), and mouse monoclonal anti-Flag (F3165, Sigma-Aldrich). Rabbit polyclonal anti-GAPDH (9545) and polyclonal anti-β-actin (A2066) were procured from Sigma-Aldrich. Immune complexes were detected with horseradish peroxidase-conjugated goat anti-rabbit (B12407), goat anti-mouse (BI2413C), or rabbit anti-goat (BI2403) immunoglobulin G secondary antibodies (Abliance).

The FXRα expression vectors were constructed by subcloning the full-length FXRα1 and exon 5 skipped FXRα isoform PCR fragments amplified by reverse transcription of HepG2 total RNA (Fig. 1*A*; forward F1: 5′-GATCGGATCCGGATCAAAAATGAATCTCATTG-3′; reverse R1: 5′-GATCTCTAGACTGCACGTCCCAGATTTCACAG-3′) directly into the BamHI/XbaI sites of the pcDNA3.2 vector (Life Technologies) in-frame with the Flag and hemagglutinin (HA) epitopes. The pHTN HaloTag CMV-neo vectors (Promega Corporation) were obtained by cloning PCR-amplified FXRα fragments (forward: 5′-GATCCCGCGGGGATCAAAAATGAATCTCATTG-3′; reverse: 5′- GATCGCGGCCGCTTAAGCGTAATCTGGAACATCGTATGG-3′) from the pcDNA3.2 vectors into the SacII/NotI restriction sites. All DNA constructs were analyzed using restriction enzymes, agarose gel electrophoresis, and Sanger sequencing. For CRISPR/Cas9-mediated genome editing of the human *NR1H4* locus, guide RNAs flanking the boundary regions of exon 5 were defined based on the hg38 version of the human genome, using the website http://crispor.tefor.net/. Guide RNAs targeting the 5′ and 3′ intronic borders of exon 5 (intron 4 forward: 5′-CACCGCAGTTGGTGAGAAAATGTCC-3′, reverse: 5′-AAACGGACATTTTCTCACCAACTGC-3′; intron 5 forward: 5′-CACCGAAGAACTTAGATGGCTTAGT-3′, reverse: 5′-AAACACTAAGCCATCTAAGTTCTTC-3′) were cloned into the Bsb1 restriction site of the pX458: pSpCas9(BB)-2A-GFP (Addgene plasmid # 48138; http://n2t.net/addgene:48138; RRID: Addgene_48138) and pX459: pSpCas9(BB)-2A-Puro V2.0 (Addgene plasmid # 62988; http://n2t.net/addgene:62988; RRID: Addgene_62988) provided by Feng Zhang (44). The pCMX-mRXRα expression vector has been described previously (45).

### Plasmid transfection and reporter assays

Human HepG2 (HCC), HeLa (cervical carcinoma), and HEK293 (embryonic kidney) cells were cultured in 10 cm plates using DMEM supplemented with 10% heat-inactivated FBS at 37 °C in a humidified atmosphere containing 5% CO_2_. NCCIT (germ cell tumor) cells were maintained in complete RPMI medium containing 10% heat-inactivated FBS and antibiotics. For transient transfections, 4 × 10^5^ cells per well were plated in 6-well plates. On the following day, transient transfections were performed using Opti-MEM reduced serum medium using the jetPEI transfection system (Polyplus). The FXRα/RXRα transcriptional activity was determined using both *Shp* and *Akr1b7*-luciferase reporter genes as using a previously described method (45, 46). Where indicated, cells were cotransfected with FXRα-expression vectors or the empty vectors (pcDNA3.2, pHTN HaloTag CMV-neo) and treated with the FXRα agonists (GW4064, 10006611; INT747, 11031), alone or in combination with the RXRα agonist LG100268 (21606) from Cayman Chemical in serum-free medium. After two days, the cells were lysed and luciferase activity was measured using a previously described method (47).

### CRISPR/Cas9 cell line editing and siRNA-mediated knockdown

CRISPR/Cas9 editing of HepG2 cells carrying the FXRα exon 5 skipping variant was generated using jetPEI transfection. Briefly, HepG2 cells were plated in 6-well plates (4 × 10^5^ cells/well) and transfected the following day using jetPEI/DNA complexes containing pX458 and pX459 plasmids. They were sorted using fluorescence-activated cell sorting (BD FACSMelody Cell Sorter, BD Biosciences) after 24 h. Isolated enhanced GFP-labeled cells were then individually plated in 96-well cell culture plates using a conditioned medium (DMEM and 10% FBS). Cellular genomic DNA expanded from individual clones was extracted and the region surrounding the CRISPR target site was amplified using PCR (forward, 5′-GATAAATTAAAAACAACAGGCTTCTCA-3′; reverse, 5′-TAATGCCACTGGGCTCCAT-3′). Sanger sequencing was used to verify correct excision.

For siRNA-mediated knockdown, HepG2 cells were transfected either with ON-TARGETplus Human U2AF2 siRNA (25 nM, L-012380–02–0005), Human hnRNPC siRNA (25 nM, L-011869–03–0005), or an NTC siRNA (25 nM, D-001810–10–05) from Dharmacon (Horizon Discovery Ltd, PerkinElmer) using INTERFERin delivery reagent (Polyplus, Illkrich, France). After 48 h, the cells were lysed for protein and RNA analysis.

### RNA extraction and gene expression analysis

Total RNAs for normal human skeletal muscle (HR-102), the small intestine (HR-306), colon (HR-311), liver (HR-314), testis (HR-401), ovary (HR-406), adrenal (HR-501), lung (HR-601), kidney (HR-901), and gall bladder (R1234118–10) were purchased from AMS Biotechnologies Europe Ltd. Mouse total RNA was isolated from adult tissues (male and female C57BL/6 animals) using Nucleospin RNA L (Macherey-Nagel SAS). Total RNA was isolated from each cell line using RNAzolRT reagent (R4533, Sigma-Aldrich). Complementary DNAs (cDNAs) were synthesized from total RNA with the MMLV reverse transcriptase and random hexamer primers (Promega Corp). Specific primer pairs used for RT-PCR and quantitative PCR assays are listed in Table 1. Real-time PCR measurement of individual cDNAs was performed using SYBR green I dye (Master mix Plus for SYBR Assay; Eurogentec) to measure duplex DNA formation using the Eppendorf Realplex system. Standard curves were generated with pools of cDNA, and results were analyzed using the ΔΔCt method. Cells were cultured with actinomycin D (5 μg/ml) in DMEM as described in the Figure 5*F* legend.

**Table 1:** Sequences of primers used for RT-PCR and RT-qPCR in this study

End point RT-PCR primers for human *NR1H4* and *mouse Nr1h4* alternative exons			Amplicon (bp)
Gene name:	Primer type (location)	Primer sequences (5′- to -3′)	
*NR1H4 all isoforms*^a^	Forward (exon 4)	CCCCAAGTTCAACCACAGAT	487/499
	Reverse (exon 6)	CTGCATGCTGCTTCACAT	346
*NR1H4 exon 5 spliced specific*	Forward (exons 4–6 junction)	GGGTGTAAAGGCTTGTTAACTG	227
	Reverse (exon 7)	TCCTGAGGCATCCTCTGTTT	
*NR1H4 exon 5 retained specific*	Forward (exon 4)	CACTGACCTGTGAGGGGTGTA	138
	Reverse (exon 5)	TCCCATCTCTTTGCATTTCCT	
*Human ACTINB*	Forward (exon 3)	CGCGAGAAGATGACCCAGATC	125
	Reverse (exon 4)	TCACCGGAGTCCATCACGA	
*Nr1h4 exon 5 spliced specific*	Forward (exons 4–6 junction)	GGCTGCAAAGGTTTGTTAACTG	123
	Reverse (exon 7)	CACTTGTCGCAAGTCACGC	
*Mouse ActinB*	Forward (exon 3)	TCATCACTATTGGCAACGAGC	92
	Reverse (exon 4)	AGTTTCATGGATGCCACAGG	


RT-qPCR primer nucleotide sequences. (h: Human-specific primers)			
Gene nname:	Forward primer sequences (5′- to -3′)	Reverse primer sequence (5′- to -3′)	Amplicon (bp)
*hACC*	CCTGACAAACGAGTCTGGCT	AGTTTGATCAGGGACTGCCG	243
*hACOX1*	AAGTATGCCCAGGTGAAGCC	AATGGTGCACGCCTTAGACA	135
*hACTIN*	CGCGAGAAGATGACCCAGATC	TCACCGGAGTCCATCACGA	125
*hADRAG1*	CTGGGACTTTGACCTTCCAGAG	AGCACTGTCTTCAGATCCATGAGC	130
*hCD36*	GCCTCTCCAGTTGAAAACCC	TGTACACAGGTCTCCCTTCT	121
*hChREBP*	TATCGACCCCACACTCACAC	TCCTCCGCTTCACATACTGG	173
*hCHRM4*	TGGCAGTTTGTGGTGGGTAA	GACCACAGGCAGGTAGAAGG	117
*hCYP7A1*	TTAGGAGAAGGCAAACGGGT	TGAGGAACTCAAGAGGATTGGC	99
*hCYP8B1*	CTGGGCAACATGCTTCAGT	ACTTGTCCTGCATAGCTGAGG	61
*hFAS*	ATCTAACTTGGGGTGGCT	CCTTGGTTTTCCTTTCTGTGCT	111
*hFXR*α *all (P1)*	ACAACAAAGTCATGCAGGGAGA	CCTGAGGCATCCTCTGTTTGT	100
*hFXR*α *e5 retained (P2)*	CACTGACCTGTGAGGGGTGTA	TCCCATCTCTTTGCATTTCCT	138
*hFXR*α *e5 spliced (P3)*	GGGTGTAAAGGCTTGTTAACTG	TCCTGAGGCATCCTCTGTTT	227
*hG6Pase*	TCAACCTCGTCTTTAAGTGGATTCT	ACTGCTTTATCAGGGGCACG	102
*hGLUT1*	GGTTGTGCCATACTCATGACC	CAGATAGGACATCCAGGGTAGC	66
*hGLUT4*	CCATCCTGATGACTGTGGCT	GATGAACCAAGGAATGGGGC	128
*hGPR68*	CCCGGTGGTCTATGTTACCG	GGCAGCGAGCAGATGTAGAA	153
*hHNF4α*	GGCAATGACACGTCCCCATC	CTCGAGGCACCGTAGTGTTT	119
*hHRH3*	TCACCCGAGCGGTCTCATA	AGAACTCGGCATAGCAGTGG	171
*hKCNN4*	CATCGGCTATGGTGACGTGG	AGCGGACTCCTTCATCTCTTTG	196
*hLDLR*	TCAACACACAACAGCAGATGGCAC	AAGGCTAACCTGGCTGTCTAGCAA	140
*hLPK*	ACGAAGGCGTGAAGAGGTTT	CCTTCTCTGCTGGGATCTCG	96
*hLRH1*	CATTATGGGCTCCTCACCTGT	TATTCCTTCCTCCACGCATTC	211
*hMCAD*	GAGTTCACCGAACAGCAGAA	AGGGGGACTGGATATTCACCA	113
*hPEPCK*	AGGTTTGACAGTGAAGGTCG	GGTGGAAGAGGCTGGTCAAT	182
*hPGC1α*	CTCCATGCCTGACGGCACCC	GCAGGGACGTCTTTGTGGCT	244
*hSCD1*	CTTGCGATATGCTGTGGTGC	TGGTGGTAGTTGTGGAAGCC	153
*hSHP*	TGGCCCAAGATGCTGTGAC	TCGGGGTTGAAGAGGATGGT	235
*hSRB1*	AGGCATTGGACAAACTGGGAAG	TGTCATCAGGGATTCAGAATAGGC	295
*hSREBP1C*	GGAGGGGTAGGGCCAACG	CATGTCTTCGAAAGTGCAATC	80
*hTBP*	CGGCTGTTTAACTTCGCTTC	CACACGCCAAGAAACAGTGA	75
*hU2AF2*	CTTTGACCAGAGGCGCTAAA	TACTGCATTGGGGTGATGTG	130

Abbreviation: RT-qPCR, reverse transcription-quantitative polymerase chain reaction.

a expected PCR fragment size: 487/499 bp (±MYTG) for exon 5 inclusion (FXRα1); 346 bp with exon skipping (FXRαse5).

For RNA sequencing, human HepG2 hepatocarcinoma cells were transfected with FXRα1 and FXRαse5 expressing vectors, and total RNA was extracted using Nucleospin RNA isolation kit (Macherey-Nagel SAS) according to the manufacturer's instructions. Sequencing was performed by the GenomEast platform, a member of the "France Génomique" consortium (ANR-10-INBS-0009). Briefly, cDNA libraries were prepared using TruSeq Stranded mRNA library prep kit for sequencing on Illumina HiSeq 4000 platform (1 × 50 bp, IGBMC). Reads were mapped onto the hg38 assembly of *Homo sapiens* genome using STAR v2.5.3a (48) and the Bowtie 2 v2.2.8 alignment tools (49). Gene expression quantification was performed from uniquely aligned reads using htseq-count v0.6.1p1 (https://pypi.python.org/pypi/HTSeq) (50) with annotations from Ensembl release 99. Only nonambiguously assigned reads were retained for further analyses and the simple error ratio estimate coefficient was used to quantify global RNA-seq sample differences (51). Read counts were normalized across libraries using the method proposed by Anders and Huber (52). Comparisons of interest were performed using the test for differential expression proposed by Love *et al.* (53) and implemented in the Bioconductor package DESeq2 v1.16.1 (http://www.bioconductor.org/packages/release/bioc/html/DESeq2.htmlCB). Log2FoldChange (Log2FC) were represented by the mean of normalized counts, and significant genes were selected using the following criteria: threshold adjusted *p*-value < 0.05 for multiple testing using the Benjamini and Hochberg method (54). Raw data are deposited at GEO with the accession number GSE279274.

### Protein extraction and Western blotting

To prepare the whole-cell lysates, cells were washed with 1 × PBS and resuspended in ice cold RIPA buffer [20 mM Tris–HCl (pH 7.5), 150 mM NaCl, 1 mM EDTA, 1 mM EGTA, 1% NP-40, and 1% sodium deoxycholate] with phenylmethylsulphonyl fluoride (PMSF, 1 mM), sodium fluoride (NaF, 1 mM), sodium butyrate (5 mM), sodium orthovanadate (Na_2_VO_3_, 10 mM), and protease complete inhibitors (Roche Diagnostics). After brief sonication on ice (2 × 20 s) and centrifugation for 30 min at 15,000*g*, 20 to 40 μg of proteins were resolved through SDS-polyacrylamide gels and transferred to nitrocellulose membranes using the Trans-Blot Turbo Transfer System (Bio-Rad). Then, membranes were blocked using bovine serum albumin (BSA) buffer [5% BSA in Tris-buffered saline-Tween 20 0.1% (TBS-T)] for 1 h and incubated with the indicated primary antibodies in BSA buffer at 4 °C, overnight. The membranes were washed three times with TBST and incubated for 60 min at room temperature in BSA buffer with horseradish peroxidase-conjugated goat anti-rabbit, goat anti-mouse, or rabbit anti-goat immunoglobulin G secondary antibodies, followed by enhanced chemiluminescence detection with Clarity Western ECL Blotting Substrate from Bio-Rad.

### GST pull-down experiments

GST control protein as well as GST-fused proteins with either the DBD or LBD of RXRα were obtained by transforming expressing plasmids into BL21 (DE3) pLysS strain-competent cells followed with 1 mM IPTG induction. GST fusion proteins were then purified by glutathione-agarose as per the manufacturer’s instructions (G4510, Sigma-Aldrich). Cold methionine-labeled FXRα proteins were generated *in vitro* using the TNT T7/T3-coupled reticulocyte lysate system (Promega Corp). Recombinant FXRα proteins (3 μl) were mixed by rocking with the glutathione-agarose bound GST proteins at 4 °C for 2 h, to perform the pull-down assay using a previously described method (55). The washed glutathione-agarose beads were boiled for 5 min in Laemmli buffer and the bound FXRα proteins were separated on a 10% SDS-polyacrylamide gel and visualized using Western blot in a ChemiDoc Imaging System (Bio-Rad).

### Gel-shift assay and EMSAs

EMSAs were performed using a previously described method (55) using the radiolabeled FXRE consensus probe (IR-1: 5′- GATGGGCCAAGGTCAATGACCTCGGGG -3′). Nuclear extracts were prepared from confluent HeLa and HepG2 cells transiently transfected either with FXRα1 isoform or exon 5-deleted FXRα variant. Briefly, cell monolayers were rinsed once with PBS 1 × and scraped in 500 μl of 10% glycerol PBS. Cells were pelleted using centrifugation at 500*g* for 5 min, washed in 500 μl buffer A (20 mM Hepes, 50 mM KCl, 1 mM EDTA, 0.25 mM EGTA, 0.15 mM spermine, 0.5 mM spermidine, 0.5 mM sucrose, 1 mM PMSF, 1 mM DTT, and 1% aprotinin) and pelleted. Pellets were resuspended in 500 μl buffer A with 0.5% Nonidet P-40 and vortexed. The cells were maintained in ice for 15 min. Homogenates were centrifuged for 10 min at 2500*g* and rinsed with buffer A without NP-40. After centrifugation at 2500*g* for 10 min to pellet the nuclei, 50 μl buffer C (20 mM Hepes, 0.45 M NaCl, 1 mM EDTA, 0.25 mM EGTA, 0.15 mM spermine, 0.5 mM spermidine, 0.5 mM sucrose, 1 mM PMSF, 1 mM DTT, and 1% aprotinin) were added, and the samples were maintained on ice for 10 min. The nuclear lysate was then centrifuged for 20 min at 13,000*g* at 4 °C, and the supernatant was placed into a fresh microfuge tube and stored at −80 °C. Nuclear extracts (5 μg) were incubated for 30 min at 4 °C in EMSA buffer containing 1 μg poly-deoxy-inosinic-deoxy-cytidylic acid (PolydIdC) and complexes were resolved by electrophoresis through 6% nondenaturing polyacrylamide gels in 0.5 × Tris-borate-EDTA [89 mM Tris (pH 8.3), 89 mM boric acid, 2 mM EDTA] electrophoresis buffer. The gels were then dried and analyzed using autoradiography in an automatic molecular imaging system (GE Amersham Typhoon).

### Glucose and triglyceride measurements

WT and CRISPR-edited HepG2 cells were plated at 4 × 10^5^ cells per well in 10% FBS supplemented DMEM with low glucose (5 mM). After 24 h, cells were serum starved for 16 h and incubated for an additional time period with normoglycemic (5 mM, low glucose) or hyperglycemic (25 mM, high glucose) DMEM without serum. Media were refreshed after 24 h, and cells were scraped at 48 h, before freezing at −20 °C in 100 μl PBS. For glucose detection, 10 μl of centrifuge-cleared cell lysates were transferred to a 96-well plate and 100 μl of glucose detection reagent (Glucose RTU, 61269, Biomerieux SA) was added. After 10 min at 37 °C, glucose content was evaluated using colorimetric assays at 505 nm using a Multiskan GO Microplate Spectrophotometer (Thermo Fisher Scientific). Intracellular triglyceride contents were measured after a 5 min incubation at 37 °C using a colorimetric enzymatic readout at 500 nm using a portion of the clarified cell lysate (30 μl) mixed to 100 μl of glycerol-3-phosphate-oxidase (GPO) according to the supplier’s instructions (Triglycerides FS∗, DiaSys Diagnostic Systems GmbH). Total protein cell lysates were measured using the bicinchoninic acid method in a 96-well plate at 562 nm (56).

### Statistical analysis

Data are expressed as mean ± SD of values obtained from multiple experiments. Graphs and statistical analyses were prepared using GraphPad Prism 8 software (GraphPad Software Inc; https://www.graphpad.com/CB). Student’s *t* test was used to compare mean values between control and tested groups, while differences between mean values of multiple groups were analyzed by two-way analysis of variance (ANOVA) with Tukey multiple comparison tests. Significance was set as ∗*p* < 0.05, ∗∗*p* < 0.01, ∗∗∗*p* < 0.001, and ∗∗∗∗*p* < 0.0001 as indicated in the Figure legends.

## Data availability

The raw data obtained in RNA-Seq in this study was submitted under Gene Expression Omnibus (GEO) accession number GEO DataSets: GSE279274. Any additional data presented in this paper are available from the corresponding author upon request.

## Supporting information

This article contains supporting information.

## Conflict of interest

The authors declare that they have no conflicts of interest with the contents of this article.
