# Supplementary material for: Alternative splicing is an FXRα loss-of-function mechanism and impacts energy metabolism in hepatocarcinoma cells
Source: J Biol Chem. 2024 Nov 26;301(1):108022. doi: 10.1016/j.jbc.2024.108022 (PMC11758954; doi:10.1016/j.jbc.2024.108022)
Supplement: Supporting information [file mmc1.docx]

**Supporting information**

**Alternative splicing is an FXRα loss-of-function mechanism and impacts energy metabolism in hepatocarcinoma cells.**

Manon Garcia^1^, Hélène Holota^1^, Angélique De Haze^1^, Jean-Paul Saru^1^, Phelipe Sanchez^1^, Edwige Battistelli^1^, Laura Thirouard^1^, Mélusine Monrose^1^, Gérard Benoit^2^, David H Volle^1,3,*^ and Claude Beaudoin^1,3,*^

^1^Université Clermont Auvergne, CNRS UMR6293, INSERM U1103, iGReD team-Volle, F-63000 Clermont-Ferrand, France ;

^2^Université de Rennes 1, CNRS UMR6290, INSERM U1305, IGDR, F-35042 Rennes Cedex, France ;

^3^Centre de Recherche en Nutrition Humaine d’Auvergne, F-63009 Clermont-Ferrand, France.

**Address correspondence to:** C. Beaudoin ([claude.beaudoin@uca.fr](mailto:claude.beaudoin@uca.fr)) and D.H. Volle ([david.volle@inserm.fr](mailto:david.volle@inserm.fr).)

**Supporting Experimental procedures**

*Ethics statement and animals.*

Mice were housed according to guidelines of the Institutional Animal Care and Use Committee at the Université Clermont Auvergne (UCA), and animal killing was carried out in compliance with standards for use of laboratory animals. Male C57BL/6 mice (12 weeks of age) were purchased from Charles River Laboratories (CRLF Ecully, France). All mice were acclimated in a temperature/humidity-controlled facility with a 12-hour lighting schedule (07:00–19:00) for one week, and fed with regular pellet rodent chow (Harlan Teklad diet, Envigo Rms Sarl Gannat, France). All mice had ad libitum access to drinking water and feed. Liver was collected postmortem from mice (n = 5/time point) at 4-hour intervals over 24 hr. The first time point was 1 hour after the light was on. Livers were rinsed with saline, frozen in liquid nitrogen, and stored at -80°C until use.

*RNA extraction and gene expression rhythm.*

Total liver RNA was prepared from frozen tissues with RNA XS Nucleospin (Macherey-Nagel SAS, France) following the manufacturer’s instruction. RNA concentrations were quantified with a Nanodrop DS-11 FX spectrophotometer (DeNovix, Wilmington DE, USA) and diluted to a final concentration of 1µg/µl. Purified RNA was stored at -80°C and 5 individual samples from each time point were used for gene expression assays. Messenger RNA of target genes were quantified by RT-qPCR as described in Experimental procedures using Table 1 specific primers targeting wild-type and exon 5 spliced *Fxrα* isoforms (*Nr1h4* exon 5 spliced specific). The mRNA levels were normalized by that of *Rsp29* (forward: 5’-TGAAGGCAAGATGGGTCAC-3’, reverse: 5’-GCACATGTTCAGCCCGTATT-3’) and *Bmal1* mRNA was assayed for ensuring circadian oscillation (forward: 5’-GCAGTGCCACTGACTACCAAGA-3’, reverse: 5’-TCCTGGACATTGCATTGCAT-3’).

**Supporting Figures**


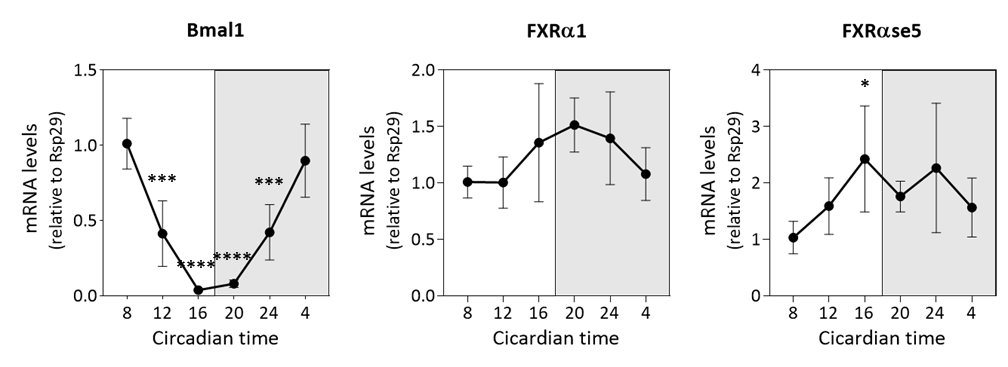


**Supplementary Figure S1: Temporal mRNA profiles of spliced exon 5 FXRα variant (FXRαse5) in the liver.** Tissues were collected from 12-old weeks male C57BL/6 mice (n=5/time point) at 4-hr intervals over 24hrs, followed by quantitative RT-PCR. The mRNA levels of each gene were normalized by Rsp29 and *Bmal1* mRNA levels were analyzed for ensuring circadian oscillation. The first time point value (8 h) was normalized to 1.0 and data are presented as means ± standard deviation (SD) with n=5 mice/time points. Difference between mean values of groups were analyzed by one way ANOVA with Tukey’s multiple comparisons test. Significance was set as **p*<0.05, ***p*<0.01, ****p*<0.001, *****p*<0.0001.


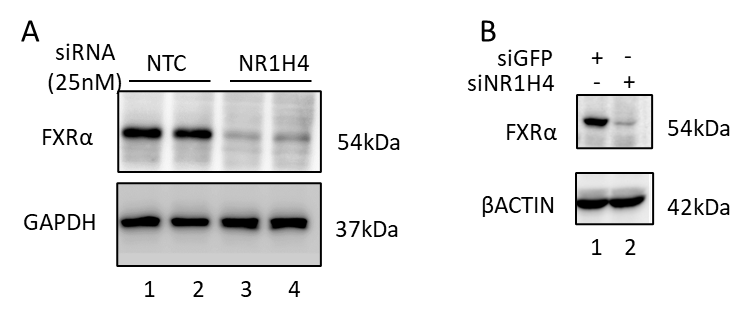


**Supplementary Figure S2: RNA interference-mediated protein knockdown validates the FXRα antibody value for Western blotting application**. For siRNA-mediated knockdown of FXRα, HepG2 cells were platted in 6 well-plates (4 x 10^5^ cell/well) and transfected the following day with ON-TARGETplus Human *NR1H4* siRNA (L-003414-00-0005) and Non-Targeting Control (NTC, D-001810-10-05) from Dharmacon (Horizon Discovery Ltd, PerkinElmer, UK) using INTERFERin delivery reagent (Polyplus, Illkrich, France). After 48 h, the cells were rinsed with PBS 1x and then lysed in RIPA buffer for whole protein cell extract analyses with a mouse anti-FXRα monoclonal antibody from Perseus Proteomics (A9033A, dilution 1:5000). GAPDH is used as a loading control for protein normalization (A). (B) Same as in panel A except that siGFP was used as control for Western blotting analysis of FXRα in HepG cell nuclear extracts as described in *Experimental Procedures*. βACTIN was used as a loading control for nuclear extract normalization.


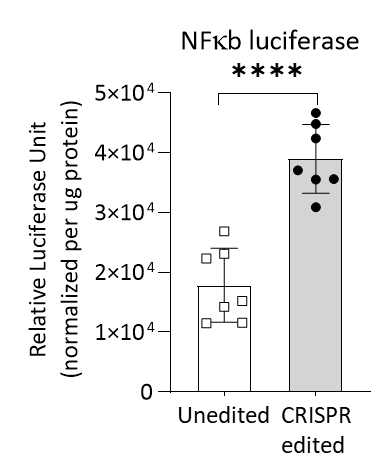


**Supplementary Figure S3: FXRα loss-of-function by exon 5 alternative splicing promotes NF-κB signaling.** Unedited and CRISPR edited HepG2 cells were plated in 6 well-plates (4 x 10^5^ cell/well) and transfected the following day with a NFκB-dependent luciferase reporter gene. Firefly luciferase activities measured after 48 h were normalized relative to protein concentrations. Results expressed as Relative Luciferase Unit (RLU) are presented as means ± SD of two independent experiments (n=7). **** *p*<0.0001 by unpaired t-test.
